# Stirring the strategic direction of scuba diving marine Citizen Science: A survey of active and potential participants

**DOI:** 10.1371/journal.pone.0202484

**Published:** 2018-08-16

**Authors:** Serena Lucrezi, Martina Milanese, Marco Palma, Carlo Cerrano

**Affiliations:** 1 TREES – Tourism Research in Economics, Environs and Society, North-West University, Potchefstroom, South Africa; 2 Studio Associato GAIA s.n.c., Genoa, Italy; 3 UBICA s.r.l., Genoa, Italy; 4 Department of Life and Environmental Sciences (DiSVA), Polytechnic University of Marche, UO CoNISMa, Ancona, Italy; Universita degli Studi di Genova, ITALY

## Abstract

Citizen Science (CS) strengthens the relationship between society and science through education and engagement, with win-win benefits. Marine Citizen Science (MCS) is increasingly popular, thanks to society’s growing interest in marine environments and marine issues. Scuba diving significantly increases the potential of MCS, thanks to the skills and behavioural properties of people who participate in the sport. To be able to exploit this potential, however, MCS needs to face challenges related to CS, to scuba diving activities and to the broader scuba diving industry. In particular, engagement and recruitment of potential volunteers, as well as retention of active participants, represent key milestones. In order to reach these milestones, information is required on current participation levels of scuba divers in MCS, as well as the motivations behind participation, and the opinions held by potential participants in MCS. This study explored different case studies and methods of data collection to provide an overview of actual and potential participation in MCS by the scuba diving community. The results show that scuba divers, whether active or potential marine citizen scientists, are well disposed towards MCS. Some barriers, however, prevent the full participation of scuba divers as marine citizen scientists. Certain barriers extend beyond the control of both divers and MCS projects, while others, such as limited access to MCS projects and poor feedback after participation, can and should be addressed. The recommendations of this research provide strategic direction to MCS, so that the broad scuba diving community can be successfully integrated into MCS. These recommendations acknowledge the important role played by stakeholders in the scuba diving industry, as well as professional intermediaries and hired experts.

## Introduction

### Citizen Science: Benefits and challenges

Citizen Science (CS), which in simple terms can be defined as the involvement of citizens in the production of scientific research [1–3], is recognised as a discipline and a crucial step towards creating or improving the relationship between society and science [4] through the communication of scientific principles, engagement and ultimately participation of citizens in the scientific process [5]. This relationship is important to establish a sense of trust by society in science, and to allow citizens to use scientific thinking in their everyday lives [5–6]. CS has been used successfully to guide and inform management, thus endorsing society democratisation [7–9]. As a result, CS has been supported by governments and scientific organisations globally. This support is reflected in dedicated governmental and nongovernmental actions worldwide, such as the establishment of CS associations.

The benefits of CS for science, society, industries, governance and policy are many and, depending on the nature and aims of a CS project, can vary in number and importance [8]. The most evident benefits of CS include the education of the public in science, and the facilitation of the scientific process through data collection by citizens over extended spatial and temporal scales [1, 10–12]. CS makes use of the exceptional skills and local ecological knowledge of people to complement traditional data collection systems [3,9]. Relevant benefits mostly accruing to participants in CS include better attitudes towards science, improved scientific knowledge and literacy, social learning, scientific thinking and the ability to use science to solve daily problems, personal growth, increased awareness and knowledge of various problems, capacity building, and a sense of pride, stewardship and proactivity [8,10–24]. CS benefits management and governance through the generation of data which can either directly inform decisions or address a specific problem [7,9]. CS is also recognised as enriching the formal scientific education experience of both teachers and school pupils by providing them with the possibility to deal with real data and scientific problems [25]. Other critical benefits of CS, which require further investigation, include the production of evidence underpinning policy-making, public participation in policy processes, positive economic impacts, and partnerships between scientists, citizens and decision-makers [8,11,26–27].

Understandably CS presents a series of challenges, which can make it difficult to implement, jeopardise its validity and reputation after it has been implemented, and make it difficult for society to understand it and appreciate it. One of the basic issues of CS relates to the ambiguity of its meaning, resulting in possible confusion among potential participants, and sometimes among scientists [9,28]. A study by Lewandowski et al. [29] has demonstrated that in the USA, the familiarity of people with the term “Citizen Science” is still low.

CS often fails to be attractive and engaging, owing to a lack of understanding of the potential market for CS, lack of inter-personal skills in liaising with communities of citizen scientists, and lack of CS promotional and marketing skills [5,8,22–24,30]. CS may be appealing to a narrow “pro-science” market, thus excluding a greater diversity of potential participants and consequently failing to achieve the main goal of broad science engagement [5,23–24]. Even when citizens possess the motivation to participate in CS, external factors such as time, distance and travel costs can easily prevent participation [22,27]. Additionally, potential citizen scientists may have little trust in their ability to really contribute to science, thus preferring not to participate in CS [22].

The data collection efforts and strict training regimens required for some CS projects can make these unattractive to potential participants or overbearing and discouraging to existing participants [31]. The monotonous nature of some CS projects, for example requiring repetitive data collection procedures and the repeated visitation of some locations, can lead to substantial drops in interest and participation [31]. During and after participation, lack of feedback and recognition to citizen scientists is a recurrent factor discouraging future participation [8,23]. After engaging citizens in the scientific process, CS projects may lose their value and utility due to the perceived trade-off between the societal and scientific benefits of CS; for example, CS is viewed as being truly successful and useful for decision-making and policy only when mutually benefiting society and science [8,25,27]. The motivations driving citizen scientists are also likely to change over time throughout participation, making it necessary for CS to find ways to keep participants engaged [24].

CS faces challenges related to the spatial and temporal scales of projects, which would require high levels of volunteer management and coordination in space and time, with impacts on the quality of the data and on the positive engagement of the participants [8]. From a method point of view, the use of technology in CS can result in unpredicted trade-offs, such as between data collection efficiency or quality and indiscriminate citizen engagement, resulting in the exclusion of important groups from participation [8,27,32]. The use of technology in CS also runs the risks of backfiring if, for example, phone applications and online sharing platforms are not properly designed, resulting in confusion and difficulties for participants [22,27].

CS faces important financial and legal challenges, including seeking funding, managing unstable sources of income over time, taking into account national and international directives on data sharing and data ownership, and risk assessment and operational safety [7,22,27,33]. These challenges tend to have repercussions on many aspects of CS from method to participants’ engagement and feedback [22].

Last, despite the large use of CS findings to create an evidence-base underpinning scientific outcomes and management decisions, the scientific validity of CS data is not trusted by some members of the scientific community, particularly those with science-centric worldviews [8,22,34]. Distrust in the accuracy and robustness of CS data often becomes the reason behind the dismissal of CS in scientific peer review, in use by other scientists, and in the formulation of policy [22,27,34]. The reliability of data collected by citizen scientists remains a fundamental issue which needs to be addressed by CS programs and project managers [34].

### Scuba diving marine Citizen Science: Potential and challenges

A number of authors have highlighted the biased attention of CS towards terrestrial environments in comparison with marine ecosystems and environments [3]. Marine Citizen Science (MCS), however, can be considered one of the stepping stones towards marine citizenship [35], and therefore should be supported. The discrepancy between terrestrial CS and MCS is starting to fade, thanks to the growing interest by the general public and citizen scientists in the field of marine science, and the consequent increase in MCS projects [23,27,36]. While this has been particularly the case for communities that live in coastal areas and are strongly associated with the sea and the ocean [22,24], the increasing accessibility of MCS projects regardless of location, knowledge and experience in marine environments has made it possible for anyone to participate in MCS [23,27,36].

Two of the reasons for the low MCS to high terrestrial CS ratio have been the inaccessibility of some marine environments, and the need for special skills in order to reach these environments, including scuba diving skills [10]. However, scuba diving is today recognised as a mass leisure activity and a tourism sector in its own right [37]. As a consequence, the scope of CS has broadened to the underwater world [18,21,38–44]. The increasing involvement of scuba divers in MCS has meant strong reductions in costs, time, equipment and logistics issues for tasks that would otherwise have been impossible to perform on the required temporal or spatial scales. By providing hands-on and engaging experiences in natural marine settings, MCS initiatives foster education on many levels among scuba divers, not only to include environmental and conservation aspects, but also safety aspects [12,44]. In addition, engaging scuba divers in MCS plays a crucial role in promoting the acceptance of and compliance with management decisions, for example those concerning marine protected areas [9,43,45]. MCS projects engaging different stakeholders in the scuba diving tourism industry can lead to new business opportunities [46], to mutual legitimation between often conflicting groups, and to better policy implementation [16]. Finally, volunteers may receive personal benefits such as: increased knowledge or improved skills, a sense of fulfilment or pride, strengthened social bonds, and increased wellbeing in terms of better physical and mental health [3,18,47–48].

Generally, the profile of scuba divers deserves attention for the potential that it possesses to be exploited in MCS. The most obvious element that renders scuba divers optimal participants in MCS is their possession of scuba diving skills, ranging from basic to professional and technical. Other useful skills associated with scuba divers’ interests include micro- and macro-photography and video-recording [10,49–54]. During their activities, scuba divers use tools including the dive computer and the bottom timer, which routinely record data for their personal dive logs; these data, such as depth, water temperature and dive time, can be used for MCS projects [44,55–56]. Scuba divers tend to travel for tourism, making it possible for them to contribute to a single international project by collecting data across different locations around the world, as well as join multiple MCS projects at the locations they visit [7,12,57]. Scuba divers participating in MCS are also likely to gain access to locations with restrictions, allowed to be explored only with scientists and for scientific purposes; this factor can contribute to motivate participation of scuba divers in MCS [43]. Similarly, dive operators supporting MCS activities can gain economic benefits from these activities, through the sale of elite package deals for marine citizen scientists or the organisation of events related to MCS [7,32,43].

Scuba divers tend to be well disposed to MCS due to their interest in the marine world, even more so than other marine users [12,18,32]. This is attributed to the basic marine environmental education that they receive during scuba diver training [12]. During training, scuba divers are educated in concepts of physics, chemistry and physiology, thus being inclined to participate in MCS that covers these topics as well [44]. Scuba divers tend to be supportive of marine education programmes [12,46]. They are also highly fascinated with and informed about marine topics in need of urgent research efforts for management and decision-making, such as shark ecology and conservation, the invasion of alien species and global climate change [4,7,46]. Therefore, it becomes easy to engage scuba divers in MCS projects revolving around these topics [4,7]. Scuba divers are valued for their local ecological knowledge, particularly for the purpose of assisting the management of marine reserves [9,42]. Recent research has showed that scuba divers represent the greatest contributing group of marine users to some MCS projects, both in terms of numbers and in terms of time [22–23]. In addition, scuba divers show interest in roles in the MCS process including not only data collection, but also data analysis and dissemination of findings [23].

Scuba divers have been involved in a variety of MCS projects. Most of these relate to the fields of environmental sciences, biological sciences, ecological and conservation sciences, oceanographic sciences and medical sciences, but some relate to archaeology, engineering and technological sciences [3] (S1 Table). Scuba divers have been supporting projects of various spatial and temporal scales, and deploying a variety of methods from sample collection to mapping and recording data with dive computers [42–43,49,56,58–60]. Scuba diving MCS has to face the same challenges as any other CS project, with some being more critical than others. The effectiveness of many CS projects relies on their ability to cover large geographical areas or operate over long temporal scales. However, the self-sustainability of these initiatives as well as their appropriate distribution in time and space are difficult to achieve because volunteers or temporarily supporting operators tend to lose interest or may no longer be able to commit enough resources, if CS fails to generate sufficient economic returns [10]. The demands of running a business like a dive resort or centre can make it difficult and not worthwhile for dive operators to either support MCS projects in addition to their duties, or participate themselves in MCS by contributing their local ecological knowledge [9].

Scuba diving can be demanding in terms of physical fitness, time, equipment use, competences and logistics. Thus, MCS projects are likely to require that divers possess a basic set of skills enabling them to collect scientific data [18,27,43,48,61]. While scuba divers appear to be keen citizen scientists and interested in some aspects of the CS process, research shows that they may not attach importance to other aspects such as the allocation of funding in MCS projects [23]. In addition, scuba divers being mostly recreational users of the marine environment, they are likely to prefer to participate in MCS at more attractive locations to merge their personal interests with their interests in MCS, to the neglect of important but less attractive locations [10]. Scuba divers who are experienced in an area could, in turn, prefer to dedicate their participatory efforts to that area and use their own baselines when contributing to MCS, with the potential to bias research [9]. The enthusiasm of scuba divers as marine citizen scientists can translate into increasing and demanding expectations from participation, such as the possibility to make use of advanced technology and equipment, better diving skills and better scientific skills [23,46,62]. Scuba divers who are actively involved in MCS are likely to be very different from scuba divers who are not. For example, they may be better educated and already possess good scientific interest and knowledge [5,18,22]. This has considerable implications for the trade-off between engaging potential scuba diving citizen scientists and retaining active participants [5,22,24]. Both active and potential scuba diving citizen scientists can be selective in their choice of and trust in various groups managing MCS projects, with effects on their decision whether to collect and share data with these groups depending on their categorisation and status [23]. Additionally, scuba divers tend to be jealous of their sport and consequently may be discouraged from sharing data for fear of losing certain privileges or having new restrictions imposed on them [22].

Generally, what underpins a solid and successful scuba diving MCS project (or any CS project) is information on its potential and existing participants or market. Despite studies looking into the profile of active scuba diving citizen scientists [3,5,18,22–24,46,48], there is still a dearth of information related to the general public opinion of potential scuba diving citizen scientists, and to the motivations, experience and feedback of scuba divers who actively participate in MCS [22–23]. Information of this sort is critical in the design, planning, promotion, engagement, recruitment and management phases of MCS projects [5,23].

### Study aim

This study is one of a number by a broader research project on sustainable scuba diving, Green Bubbles RISE (www.greenbubbles.eu), funded by the European Commission. The four-year project, which commenced in 2015, focuses on the sustainability of the scuba diving industry in the Mediterranean regions by means of case studies, but it also involves secondary case studies located outside Europe for comparison. While the main aim of the project is to carry out research that can assist the scuba diving industry in achieving sustainability goals, the project is divided into a number of main objectives. One is to enable the scuba diving industry to properly engage divers in MCS and give win-win benefits to divers, diving operations and the scientific community. To achieve this objective, entities involved in the project had tasks ranging from the development of new and improved CS technology, which would appeal to the diving industry, to the launch of initiatives of collective MCS project management by the diving industry and scientists. These tasks, however, need to be underpinned by relevant information on the opinions of potential participants and on the experience of existing scuba diving citizen scientists.

Therefore, this study aimed to evaluate the opinion of scuba divers on MCS. To achieve this aim, the experience and opinion of active and previous scuba diving marine citizen scientists were explored, as well as the opinion of potential participants in MCS. The prospect of this study was to generate the necessary knowledge to enhance the strategic direction of MCS in increasing public interest and participation, retaining existing scuba diving citizen scientists and re-engaging previous citizen scientists who have abandoned the prospect of future participation.

## Method

This study was approved by the Faculty of Economic and Management Sciences Research Ethics Committee (EMS-REC) at the North-West University under the ethics code EMS2016/11/25-02/22. No private personal information was asked from the participants in the study. The data were handled according to laws on privacy and oral consent was provided by the participants before the study. Participants were able to leave the research at any point during the study.

### Survey design and structure

The research followed a quantitative, non-experimental and descriptive method of data collection, using a structured questionnaire survey as the measuring instrument (S1 and S2 Questionnaires). The survey was designed by members of the Green Bubbles project who were directly involved in covering the CS aspects of the project.

The survey was structured into a general section followed by two separate sections; the scuba divers needed to compile either one or the other, depending on whether or not they had previously participated in CS. The general section contained questions capturing the demographic profile of the participants (e.g. gender, age, education level, nationality and occupation) and the scuba diving history of the participants (number and type of scuba diving certifications held, number of years diving, number of dives logged and average number of dives logged annually). At the end of this section, the participants were provided with a definition of CS based on Bonney et al. [1]. CS was described as “any involvement of volunteer participants in the generation of scientific knowledge, resulting in reports supporting management decisions and scientific publication.” Participants were then asked whether or not they had ever participated in CS. Those who answered positively were invited to move on to the next section of the survey, while those who answered negatively were asked to complete a separate section of the survey.

Scuba divers who had participated in CS were asked to indicate the number of MCS projects they had been involved in, the number of times they had been involved in these projects and for how long. Then, they were asked to provide the details of the latest MCS project they participated or were participating in. These details included project name, location, number of times they participated and for how long, project subject, method of recruitment, their role and duties in the project, number of volunteers involved, type of data collected, materials and methods for data collection, type of environment where data were collected, spatial and temporal scales of the project, and any incentives received for participating. The scuba divers were asked to indicate their reasons for participating in MCS, by agreeing or disagreeing (using a Likert scale where 1 = disagree and 3 = agree) with statements reflecting known motivations reported in the literature. Finally, they were asked to indicate their level of satisfaction (using a Likert scale where 1 = unsatisfied and 3 = satisfied) with statements related to participation in MCS. These statements were also drawn from relevant MCS literature.

The section of the survey dedicated to scuba divers who had never participated in CS covered questions exploring their potential involvement in MCS. First, scuba divers were asked whether or not they had ever been interested in MCS. Those who answered positively were asked in more detail which subjects were of interest to them and why they found MCS interesting in the first place, through a series of Likert scale questions (1 = disagree to 3 = agree). Those who answered negatively were asked why MCS was not attractive to them, using a similar Likert scale system. Then, scuba divers were asked whether they would be interested in participating in an MCS project. Those who would consider it were asked why they had never participated before and what would convince them to participate, always using a Likert scale series of items. In a similar way, scuba divers who would not consider participating in MCS were asked why (they were given the possibility to say that it was for the same reasons why MCS was not attractive to them in the first place), and whether there would be any factors stimulating their interest in participating.

### Survey administration

The survey was administered in two different ways. The first included the distribution of hard copies using random sampling techniques at selected case studies, while the second included the promotion of the questionnaire survey online. As will be discussed, the results were evidently influenced by the method of data collection.

#### Field survey at selected case studies

The first method of data collection was characterised by the distribution of hard copies of the questionnaire at selected case studies. The Green Bubbles project revolves around the scuba diving industry of the Mediterranean Sea. Based on this focus two case studies were selected. The first is the marine protected area of Portofino, Italy, located in the north-western Mediterranean basin. The second is the Republic of Malta in the southern Mediterranean basin, including the two islands of Malta and Gozo. Both of these case studies are among the most important scuba diving destinations in Europe, hosting a large number of visiting scuba divers annually [62–63]. In order to allow a comparison between the Mediterranean case studies and other scuba diving destinations in developing countries, Green Bubbles included a third case study, namely the village of Ponta do Ouro in the Ponta do Ouro Partial Marine Reserve (PPMR), located in southern Mozambique at the border with South Africa. The coastal village is an important scuba diving tourism destination for South African and international visitors [62].

Each case study hosts a large number of dive centres over a small geographical area, thus requiring no more than two fieldworkers to collect data. The questionnaire surveys were distributed in both Italian and English at the Italian case study, and in English at the Maltese and Mozambican case studies. Sampling started in 2015 in Mozambique, where it took place every second day throughout June. Fieldworkers then moved to Italy, where sampling occurred every second day throughout August 2015. Sampling ended in Malta in 2016, where it took place every second day throughout October. During sampling, the trained fieldworkers approached all the local dive centres at the case studies regularly, haphazardly inviting scuba divers either preparing for or returning from dive trips to participate in a brief 10-minute survey. Scuba divers who agreed to participate were asked to compile the survey at the dive centre, to ensure that they could be assisted if needed. The fieldworkers put in similar sampling efforts and were in possession of equal numbers of questionnaire surveys (150) for each case study. However, participation rate was not necessarily expected to be the same among case studies.

#### Online survey

The same survey was prepared in Google Forms and promoted online using a mixture of snowballing and convenience sampling strategies, in a similar fashion to what is described by Martin [5] and Martin et al. [22]. The survey, which exclusively targeted scuba divers, was launched through the social media platforms (Facebook and Twitter) of the Green Bubbles project, as well as those of the participating entities (eight) and researchers in the project, including personal pages and group pages. The online survey was also emailed, accompanied by an introductory letter, to a number of dive operators and diving magazines in those countries where participating entities in the project were either located or working (Italy, the Netherlands, Malta, Turkey, South Africa, Mozambique and the USA). The survey was open for one year from November 2016 to November 2017. Unlike the field survey, the online survey offered an incentive. Participating scuba divers were invited to provide their email address at the end of the survey, in order to take part in a lucky draw for a small prize, which consisted of a beach bag. While the controversy surrounding the use of incentives in social science research has been pointed out [5], a small incentive such as the one used here was unlikely to have a particular effect on the sampling outcome.

### Data analysis

Due to the two different methods of data collection, the final data sets were treated separately where possible. The analysis mostly compared variables across all case studies, including the three physical case studies and the online survey. However, in the case of non-significant comparisons across the case studies, the data were pooled and analysed together.

All the analysis was performed using the software Statsoft Statistica (Version 13.2, 2016). All graphs were created using GraphPad Prism (Version 5.03, 2010). The data were analysed using descriptive, univariate and multivariate techniques. First, the profile of the participants and their main answers were analysed with descriptive statistics, breakdown statistics, and frequency tables. When analysing the profile of the participants, results from normality tests (Chi-Square and Kolmogorov-Smirnov) showed that continuous variables, such as age and number of years diving, had non-normal distributions. For the categorical variables, there were no cases where scores were greater than 90% within a category. Therefore, the demographic profile of the participants was compared between case studies (including the three physical case studies and the results from the online survey) by performing either Kruskal-Wallis (for non-parametric variables) tests or one-way ANOVA (for parametric variables), where appropriate. Correlational relationships between some of the variables (e.g. age, number of years diving and total number of dives logged) were calculated using non-parametric Spearman correlations.

Variables in the scuba diver profile which were significantly different across study sites included age, total number of scuba diver certifications held, number of years diving, number of dives logged annually, and total number of dives logged. These variables were included as co-variates in a General Linear Model (ANCOVA). The model was used to assess the variation of responses by people who had never participated in CS across the case studies. Binomial logistic regressions were executed to establish whether scuba diver profile variables (e.g. age, total number of dives logged) influenced previous participation in CS, interest in MCS, and willingness to participate in MCS.

Since the number of scuba divers who had previously participated in MCS was relatively low, the details provided by the scuba divers and their experience as participants in MCS were mainly analysed using descriptive and breakdown statistics, and represented graphically.

## Results

### Demographic and scuba diving profile

A total of 362 scuba divers participated in the survey (80% pooled participation rate), of whom 112 came from the Portofino case study, 87 from the Ponta do Ouro case study, 56 from the Malta case study, and the remaining 107 from the online survey. A total of 25 dive centres were sampled at the three case studies of Portofino, Malta and Ponta do Ouro.

The demographic and scuba diving profile of the participants is outlined in Table 1. The participants were mostly men in their late thirties or early forties. Generally, the highest level of education of the participants was graduate or postgraduate; in Malta and Ponta do Ouro, however, the highest level of education was mainly a high school diploma, followed by an academic degree. Participants with undergraduate and postgraduate education had received degrees in a variety of subjects, with marine science, engineering, economics and medicine being the most common. Evidently, the online survey attracted a large number of scuba divers with a background in marine science. The majority of the scuba divers in Portofino were Italian; those in Malta came from a variety of countries, especially the UK, Italy and Germany. The scuba divers in Ponta do Ouro were mainly South African, and those who participated in the online survey were mostly from the USA, Italy, South Africa and the Netherlands. The participants were employed in a number of professions, with scuba diving, engineering, management, academia and medicine being most mentioned. Many of the participants working in scuba diving were based in Malta, while most academics were participants in the online survey.

**Table 1:** Demographic and scuba diving profile of the participants (N = 362).

	Portofino	Malta	Ponta do Ouro	Online	All
**Gender**	75% male	64% male	49% male	69% male	65% male
**Age (mean±SD)**	40±12	37±12	36±13	43±13	39±13
**Education level**	Graduate, postgraduate	Diploma, graduate	Diploma, graduate	Postgraduate, graduate (marine science)	Graduate, postgraduate, diploma
**Occupation**	Paid work	Paid work (scuba diving)	Paid work	Paid work (academia)	Paid work
**Working in scuba diving?**	6%	43%	3%	7%	12%
**Grew up**	79% inland	71% inland	82% inland	61% inland	73% inland
**No. scuba diving certifications (mean±SD)**	8±8	11.5±12	3.5±5.5	8.5±7	7.5±8
**No. basic scuba diving certifications (mean±SD)**	3±2	3±1	2±1	3±1.5	2.5±1.5
**No. professional scuba diving certifications (mean±SD)**	2±2	4±4	3±5	2±1.4	3±3
**No. specialty scuba diving certifications (mean±SD)**	4±3.5	5±6	3±3.5	3±4	4±4
**No. technical scuba diving certifications (mean±SD)**	3±2.5	4±4	1.5±1	3±2	3±2.5
**No. dry certifications (mean±SD)**	1.5±1	3±2.5	2±2.4	2±2	2±2
**No. other scuba diving certifications (mean±SD)**	1±0.5	1±0.5	0	1.5±1	1.4±1
**Years diving (mean±SD)**	12±10	12±8	7±6	14±12	12±10
**Dives per year (mean±SD)**	38±44	202±200	33±87	55±78	68±117
**Dives logged (mean±SD)**	465±909	1278±1 730	236±684	533±868	569±1 088

Generally, the participants were experienced scuba divers, with an average of eight certifications, about 10 years of diving history, logging over 50 dives per year, and having logged over 500 dives in total (Table 1). The experience of the divers is also reflected in the average number of professional, specialty and technical certifications held (Table 1). The variables of age, total number of scuba diving certifications held, number of years diving, number of annual dives, and number of dives logged all had significant positive correlations with each other (Spearman *r* = 0.17 to 0.82, *P* < 0.05). From the comparison between case studies, it is clear that the participants in Malta possessed the most experience, while those in Ponta do Ouro possessed the least.

### Participation in MCS

A total of 60 scuba divers (17%) claimed to have been involved in CS projects. The greatest proportion of people involved was among participants in the online survey (27%), followed by scuba divers in Portofino (20%) and Malta (20%), with the scuba divers in Ponta do Ouro being the least involved (2%). Since the number of scuba divers participating in CS was comparatively small across the case studies, data on participation are reported collectively.

Participation in CS was significantly influenced by the demographic and experience profile of the scuba divers. Older divers tended to have participated in CS in greater proportion compared with younger divers (Logistic regression Wald test = 4.91, *P* < 0.05). Professional and advanced divers had also participated in greater proportion compared with basic divers (Logistic regression Wald test = 19.60, *P* < 0.001). Similarly, people who had been diving for longer (Logistic regression Wald test = 18.85, *P* < 0.001), had logged more dives (Logistic regression Wald test = 10.98, *P* < 0.001), and were diving more frequently annually (Logistic regression Wald test = 9.10, *P* < 0.01) had been involved in CS in significantly greater proportion compared with people who had been diving less.

The scuba divers had participated in an average of 2.3 MCS projects (SD = 3.2; SE = 0.42; range = 1 to 20), although over 60% of them had participated in a single project only. The scuba divers claimed to have participated up to 100 times in a given project, although 37% of them had only participated once. While the commitment to an MCS project was for a single day for 23% of the participants, 13 people (22%) claimed to have been committed to MCS projects for years ranging between one and ten, and the rest (55%) had been committed for a period ranging from days to months.

#### Details of MCS projects

When asked to provide details of the last or current MCS project they participated in, the scuba divers listed projects distributed across 14 countries including Italy, USA, Mexico, Spain, Malta and more tropical destinations like Ecuador and Maldives. The scuba divers listed a variety of projects, mostly mentioning national and international long-term ones including Reef Check (18 scuba divers), Diving Safety Laboratory (9 scuba divers), and Project AWARE litter clean-up and monitoring events (4 scuba divers). They also mentioned more local, long-term or once-off initiatives relating to marine species monitoring, particularly of large animal species (e.g. manta rays). The majority (75%) confirmed biology, environmental sciences, ecology and conservation as the main fields of the projects they participated in, and 15% mentioned medical and safety research projects. The data they collected were biological and ecological, related to either fauna (55%) or flora (40%); environmental data (33%); and in the case of medical research, physiological (18%).

The scuba divers had heard about projects in various ways, particularly through word of mouth (25%), from an organised group (25%), from the internet (17%), and from other sources such as universities (6%). Participation mostly lasted between a single day (25%) and a few months (17%), although in five cases it was ongoing, and in eight cases it lasted for years. The scuba divers participated mostly as active volunteers (72%), although some were coordinators (13%) and training scientists (8%). About a third (27%) of the scuba divers went through a selection process in order to participate. The type of preparation received prior to data collection was characterised by brief instructions (38%), special training (30%), and basic training (13%). Group sizes of participants normally did not exceed a few tens of scuba divers (57%), although in 32% of the cases they reached hundreds of people.

The scuba divers tended to collect data with other volunteers and scientists (80%), and not on their own. The materials provided by the organisers to the divers included data sheets, equipment such as quadrats, tape measures and GPS, and software or applications. Dive centres supporting data collection operations offered their infrastructure, such as vessels and internet connection. The scuba divers used their own diving equipment, dive computers and cameras. Generally, they had to travel to reach a destination for data collection, with half of them travelling up to 150 km, and the remaining covering distances of up to 10 000 km to participate in an MCS project (mean = 913 km; SD = 1 553; SE = 229; range = 0 to 10 000 km). Although 18% did not have to spend any money for participation, 35% spent up to the equivalent of USD150, and the rest even more.

The type of data collected included mostly written numbers and notes (60%), images and videos (38%) and samples (25%). The data were collected principally in shallow water, corresponding to the maximum limit of 18 m allowed for PADI Open Water Divers (63%) and/or deep (43%) water, corresponding to any depth below 18 m. Aside from data collection, other duties of the participants included mainly data entry (25%) and data reporting either verbally or through the web (23%). In 17% and 13% of the cases, respectively, data analysis and writing were also performed by the scuba divers. Nearly all (83%) scuba divers claimed that they did not receive any compensation for their participation. Those who did, received some sort of gift, such as free dives or money.

#### Motivations and satisfaction of participants in MCS

**Fig 1:**
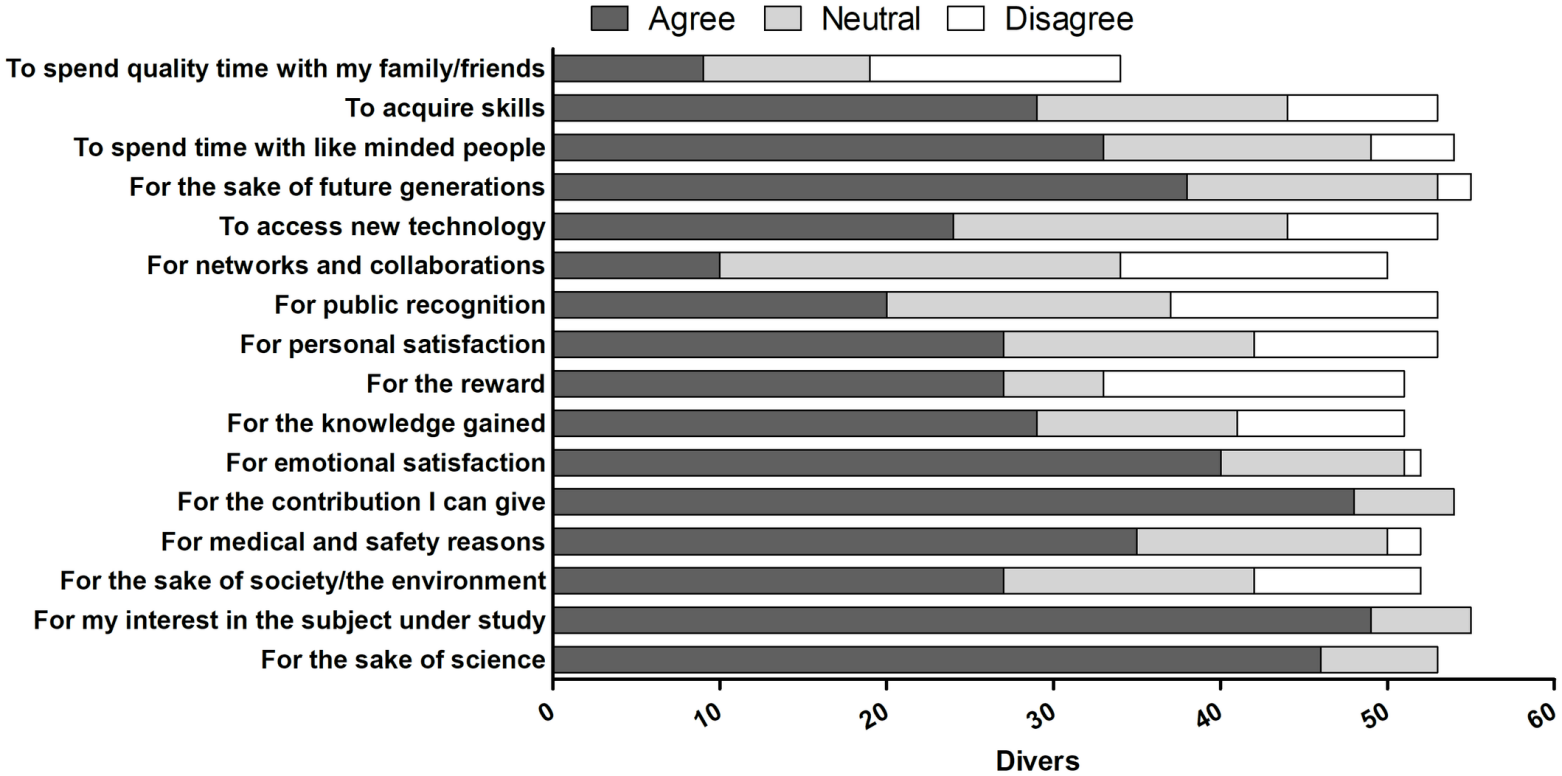
Main motivations of scuba divers who have participated in MCS (n = 60).

Scuba divers mainly participated in MCS for their interest in the field under study (82%), for the contribution they could make (80%), and for the sake of science (77%; Fig 1). Other strong motivations included emotional satisfaction (67%), generational equity (63%), medical and safety reasons (58%), and spending time with like-minded people (55%). The least important reasons included spending quality time with family and friends (15%), building networks and collaborations (17%), and receiving public recognition (33%; Fig 1). Receiving an award was considered a motivation by 45% of the divers who participated in MCS. However, this item was also disagreed to be a reason to participate by the greatest proportion of divers, specifically 30% (Fig 1).

**Fig 2:**
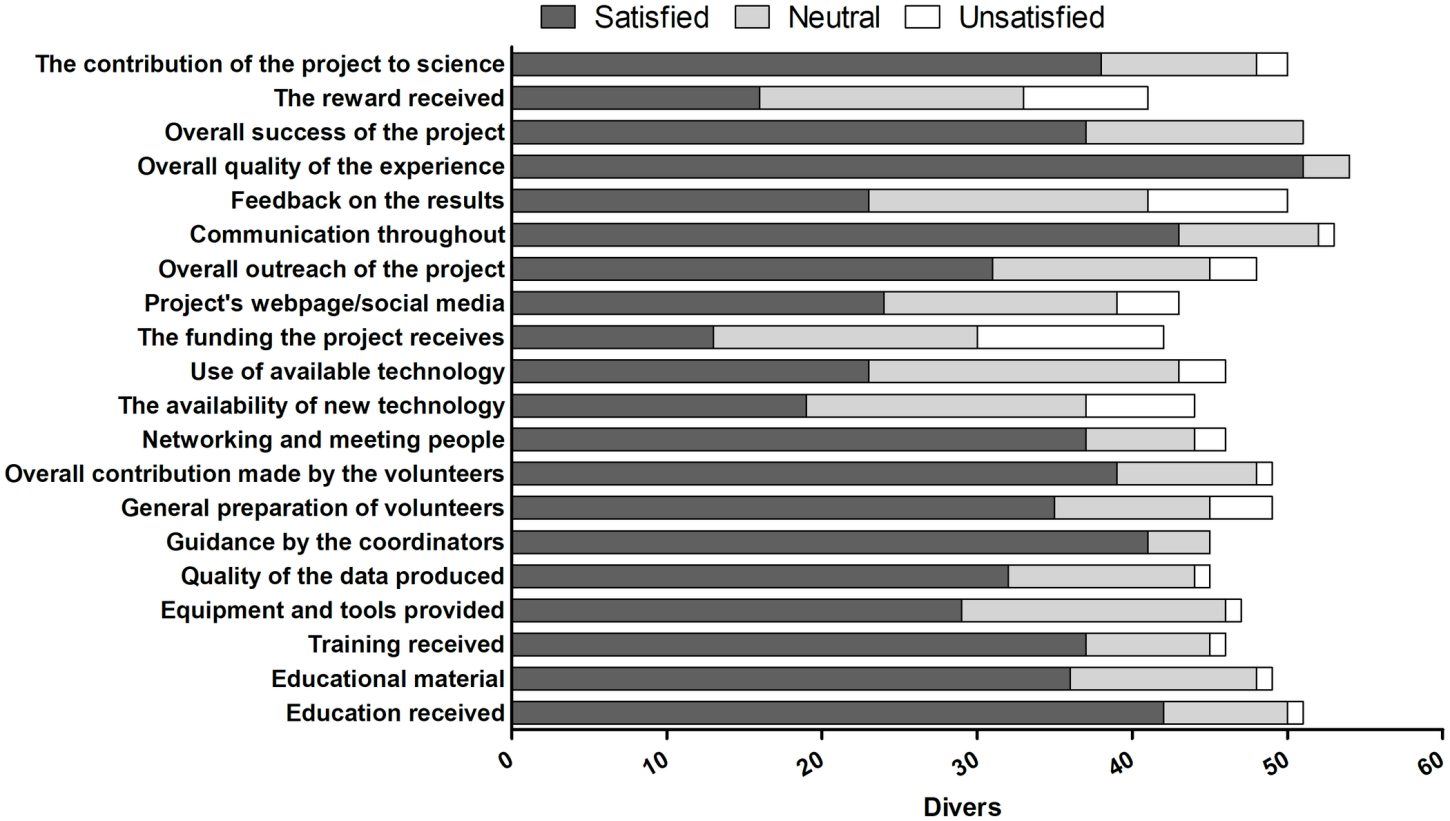
Satisfaction levels of scuba divers with different aspects of their MCS experience (n = 60).

Scuba divers were most satisfied with the overall quality of the experience (85%), the communication throughout (72%), the education received (70%), and the guidance of the coordinators (68%; Fig 2). Other satisfying elements included the overall contribution made by the volunteers (65%), the contribution of the project to science (63%), the overall success of the project (62%), the networking (62%), and the training (62%) and educational material received (60%). The scuba divers were least satisfied with the funding received by the project (22%), the availability of new technology (32%), and the feedback on the results (38%). Some divers were dissatisfied with the reward received (13%), although this proportion was substantially lower than the proportion of divers who were satisfied with it (27%).

### Attitudes of non-participants in CS

**Fig 3:**
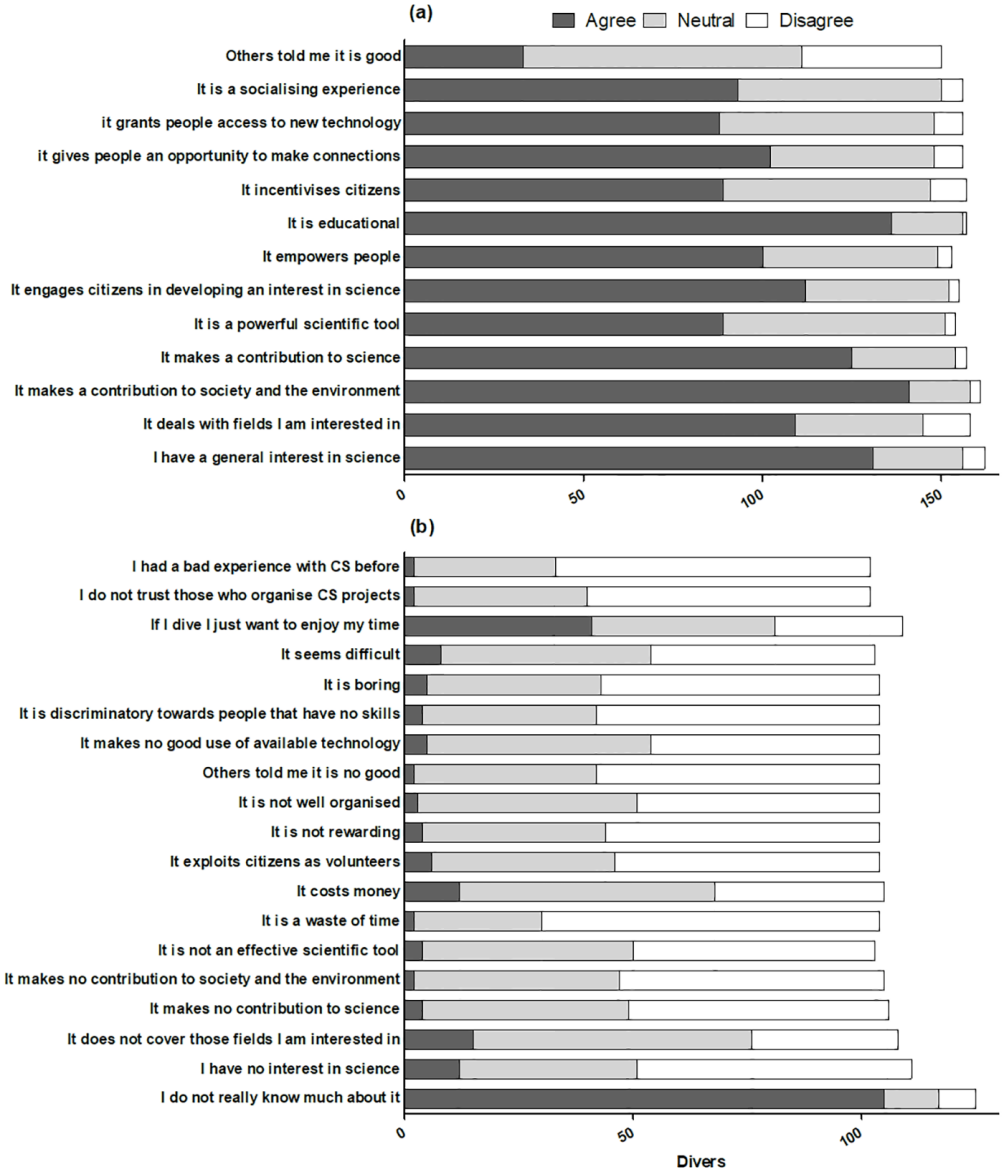
Reasons of interest (a) and lack of interest (b) in MCS among scuba divers who have never participated in MCS (n = 302).

**Fig 4:**
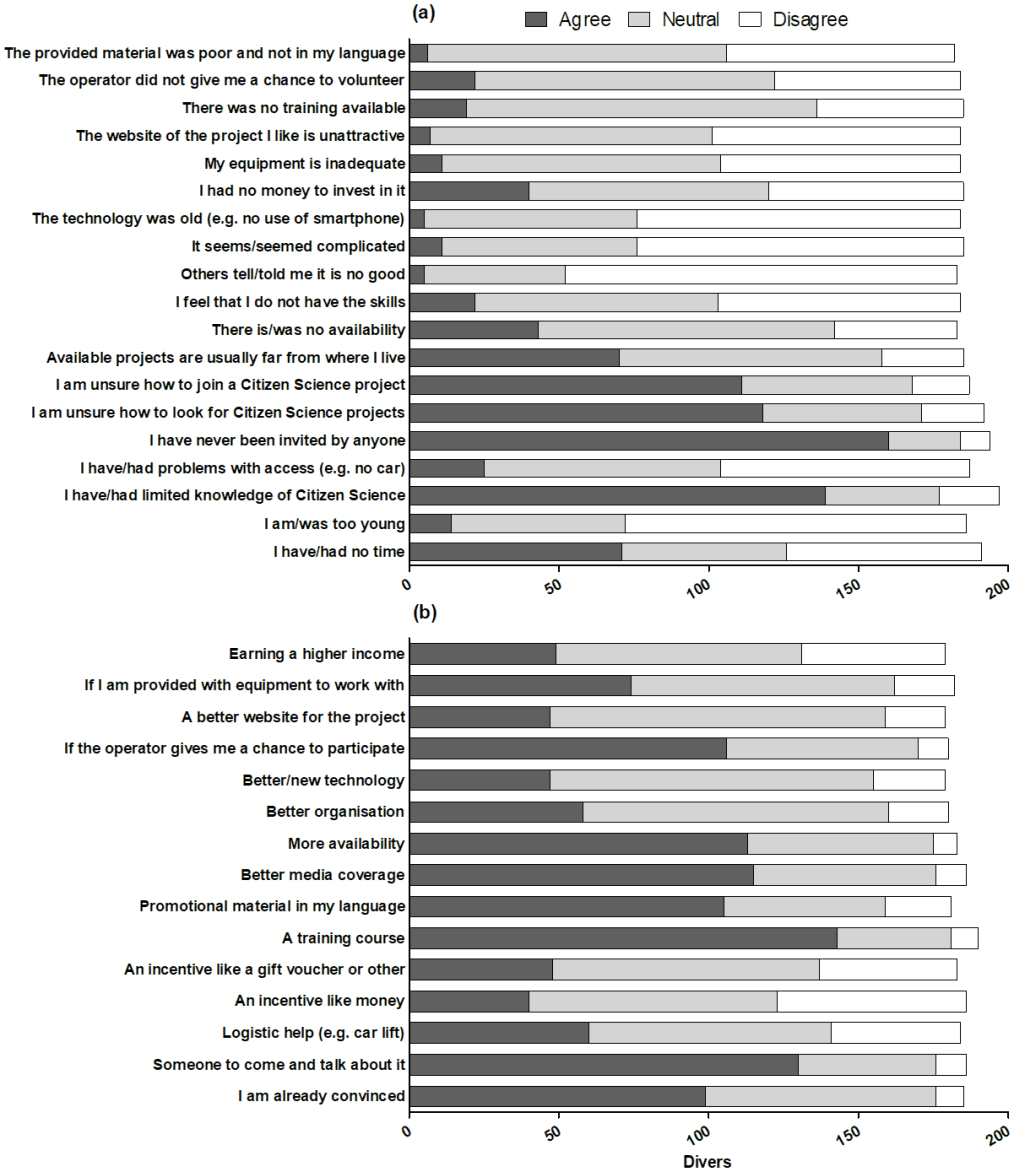
Reasons why scuba divers interested in participating in an MCS project have never participated previously (a), and potential incentives for future participation (b) (n = 204).

**Fig 5:**
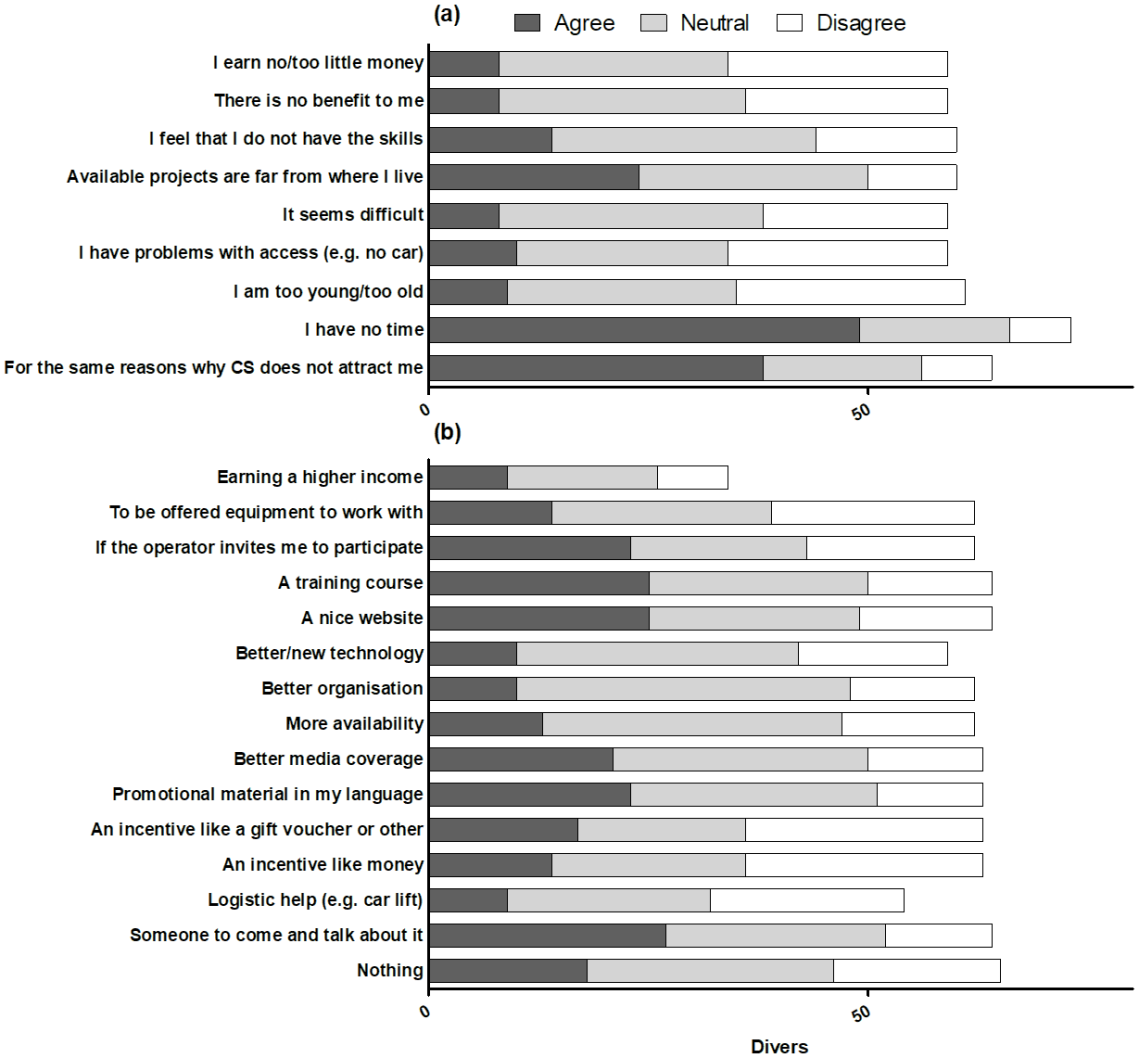
Reasons why scuba divers would not consider participating in an MCS project (a), and potential incentives for future participation (b) (n = 83).

An overview of the attitudes towards MCS among scuba divers who never participated in CS is displayed in Table 2, as well as Figs 3, 4 and 5. Overall, the ANCOVA tests comparing attitudes of non-participants in CS across case studies had non-significant results. Therefore, all responses tended to be treated as a whole. In answer to the question whether they had ever been involved in CS, 83% of the scuba divers answered no (Table 2). The percentage of people who had never participated in CS ranged between 73% (online survey) and 98% (Ponta do Ouro). Among these people, however, over half claimed to have been interested in MCS, particularly in the field of biology, ecology and conservation (Table 2). Other interests included medical research (particularly in Portofino), and technology and engineering. Interest in MCS was significantly greater among scuba divers with professional and advanced certifications compared with divers with basic certifications (Logistic regression Wald test = 8.16, *P* < 0.05). When asked more specifically why MCS was of interest to them, scuba divers agreed that MCS makes a contribution to society, to the environment and to science (85%); that it is educational (82%); that they had a general interest in science (79%); and that MCS engages citizens in developing an interest in science (68%; Fig 3a). Scuba divers who had never been interested in MCS agreed that this was largely because of their lack of knowledge regarding MCS (81%), and because they just wished to enjoy themselves while scuba diving (32%; Fig 3b).

**Table 2:** Interest in MCS among the divers who have never participated (n = 302).

	Portofino	Malta	Ponta do Ouro	Online	All
**Not involved in CS**	84%	80%	98%	73%	83%
**Ever been interested in MCS**	44%	60%	42%	78%	55%
**Biology, ecology and conservation**	78%	89%	75%	87%	82%
**Medicine, health and safety**	56%	15%	17%	25%	29%
**Technology and engineering**	34%	19%	36%	39%	34%
**Social sciences**	20%	22%	19%	13%	18%
**Would be interested in participating in an MCS project**	68%	69%	52%	83%	68%

When asked whether they would be interested in participating in an MCS project as divers, nearly 70% of the participants who had never been involved in CS answered positively (Table 2). This interest was greater among divers with more professional and advanced certifications (Logistic regression Wald test = 7.54, *P* < 0.05). Participants in the online survey (83%) were the most interested, while scuba divers in Ponta do Ouro were the least interested (52%; Table 2). Scuba divers who were interested in participating in an MCS project had not participated in the past due to never being invited by anyone (78%); possessing limited knowledge of CS (67%); being unsure how to look for (57%) and join (54%) a CS project; not having any time (34%); and living too far (34%; Fig 4a). These divers agreed that good incentives to participate in MCS would include a training course (69%); someone talking to them about MCS (63%); more availability (55%) and better media coverage (56%) of MCS projects; and scuba diving centres and operators giving them a chance to participate in MCS (51%; Fig 4b). They also gave minimal importance to money (19%) and gifts (23%) as incentives that would convince them to participate (Fig 4b).

The scuba divers who were not interested in participating in an MCS project agreed that this was because of a lack of time (59%), and for the same reasons that they had never been interested in MCS (46%), namely lack of knowledge and wanting to enjoy themselves while scuba diving (Fig 5a). Some of these people would consider participating if someone talked to them about MCS (33%); if they received a training course (30%); if MCS was marketed through a nice website (30%) and good promotional material (28%); if the operator at their dive centre invited them to participate (28%); and if MCS received proper media coverage (25%; Fig 5b). Also in this case, divers did not give too much importance to incentives like money (17%) and gifts (20%) as factors stimulating potential participation.

## Discussion

This study investigated the participation of scuba divers in MCS, as well as their opinion of MCS and that of potential participants. The results are discussed here to guide the strategic direction of scuba diving MCS and, in particular, to propose ways to increase public interest and participation in MCS, retain existing scuba diving citizen scientists and re-engage previous citizen scientists who have abandoned the prospect of future participation.

### Scuba diver profile and implications for MCS

The scuba divers who participated in this study were mostly well-educated middle-aged men, which comprises the majority of the scuba diving market worldwide [64]. While the online survey attracted scuba divers with a background in marine sciences, the overall population in this study tended to be educated and possess professions in the STEM (science, technology, engineering and mathematics). A number of the participants were also employed in the scuba diving industry. The knowledge and involvement of scuba divers in STEM can make them ideal candidates for participation in a number of MCS projects. Studies have demonstrated that scuba divers represent a special group of marine citizen scientists, who wish to dedicate more time and effort to the scientific process compared with other marine users [22–23]. A scientific disposition among scuba divers, who also tend to possess higher educational qualifications, makes it easier for managers of MCS projects to engage this group [32]. The results, however, show that most participants had never been involved in CS, indicating that other factors played a role in influencing participation and interest.

The analysis of the data shows that scuba diver age was the single demographic variable influencing participation in CS, and that no demographic variable influenced interest in MCS. This implies that older divers were more likely to have participated in CS compared with younger people, but that the latter were not necessarily uninterested in MCS. Therefore, age does not seem to prevent the consideration of MCS in scuba divers, although other studies point to the contrary [29,32]. Scuba diving is a male-dominated sport [64] and the results of this study reflect this reality. Females have been demonstrated to show a greater propensity towards participation in MCS [23,29,32]. Therefore, it could be argued that the limited participation in CS depicted in this study is due to gender bias in the sample, among other factors. However, the general enthusiasm of scuba divers, including males, towards MCS [12,22,43] seems to exclude the possibility that gender could have ultimately determined the outcome of this research.

The scuba diving experience of the members in this study had a strong role in determining participation in CS. Indeed, scuba diver experience was positively correlated with age, which positively influenced participation in CS. However, experience, particularly the number and type of certifications held, also influenced a general interest in MCS and the interest in future participation in MCS. These results indicate that advanced and professional divers are likely to be a strong pro-MCS group, and there are a number of possible interpretations of this finding. One reason could be that some scuba diving MCS projects require above-average scuba diving skills in order to carry out specific data collection tasks [3,65]. Experienced and professional scuba divers tend to also possess high levels of local ecological knowledge and other knowledge pertaining to the scuba diving sport (e.g. decompression, diving with mixed gases, diving below recreational depths); these qualities make them highly sought after for MCS projects in various fields [9,50]. In addition, it is possible that professional divers exhibit particular interest due to the financial and marketing potential of MCS [46]. Last, experienced scuba divers tend to occupy a special spot in what is called the scuba diver specialisation continuum. According to the diver specialisation theory, adapted from the recreation specialisation theory [66–67], scuba divers who acquire more skills and knowledge tend to show greater interest and higher involvement in scuba diving activities, also seeking different, less ordinary and more stimulating experiences compared with novice divers [68–70]. Depending on their nature, MCS projects can represent new, unique and non-conventional experiences, thus attracting specialised divers [43]. It must be noted that many MCS projects today aim to be all-inclusive and simple-tasked [18,50] and in that case the prerequisite of exceptional skills in order to participate becomes only a perception among potential participants [22]. This can have detrimental consequences on the engagement of a broad scuba diving market in MCS. The ideal scenario for the future engagement of scuba divers in MCS sees a mixture of projects addressed to different markets in the scuba diving community. This scenario is becoming a reality, although projects that could be stimulating for experienced divers still remain a minority at present (S1 Table).

### Lessons from participants in MCS

The results of this study show that the proportion of scuba divers who were participating or had previously participated in MCS was relatively low (17% of the total sample). In addition, this small group appeared to have generally limited experience of MCS, having participated in no more than two MCS projects on average and mostly having participated in a single project. While these results point to little familiarity with MCS, the small sample who claimed to have participated in MCS was somewhat varied in frequency of participation and level of dedication, with some people having committed to a given project over the long term and more than once. Dedication stretched beyond time to include a commitment to cover large distances and spend money in order to participate. The willingness to pay in order to participate in MCS is comparable to other positive attitudes which tend to characterise specialised scuba divers. Research on specialisation has shown that as divers become more experienced, they are likely to grow more attached to the diving activities and be willing to invest time, money and other resources to safeguard these activities and the resources that they depend on [68]. The motivations to participate in MCS were mostly intrinsic and altruistic, and excluded normally recognised motivations such as public recognition [3,23], demonstrating the apparently selfless nature of the typical scuba diver participating in MCS. These findings support research describing the commitment (both actual and potential) and enthusiasm of scuba divers as marine citizen scientists [22–23]. Finally, the geographical span of participation was impressive, highlighting the relevance of scuba divers as travelling marine citizen scientists [7,12,57]. Some of the scuba divers who had previously participated in MCS agreed that they did so partly for the reward. However, this was one of many overriding motivations to participate in MCS.

The main fields of participation in MCS by the scuba divers, namely those of marine biology, ecology and environmental sciences, reflect the general structure of MCS participation by scuba divers as reported in the literature [3] (S1 Table). Scuba divers tended to have participated more in popular international projects. This finding demonstrates the plasticity of international MCS projects which tend to have standardised protocols of participation, thus increasing the likelihood of people participating easily no matter where. However, a good proportion of the scuba divers also mentioned more local MCS projects, particularly associated with research on mega fauna (e.g. manta rays). This result points to the relevance of potential trade-offs between international MCS projects, which possess advantages such as popularity and accessibility, and smaller-scale projects, which have advantages such as a specific focus on charismatic and attractive species. While different types of MCS projects have their strengths and weaknesses, maintaining diversity in such a variety is important in order to engage and retain a broad scuba diving market with different needs and interests.

Scuba divers participating in MCS had mostly been engaged through word of mouth and organised groups, with the internet playing a nearly negligible role in promoting MCS. Thus, while communication and simple forms of engagement regarding MCS can be effective through the World Wide Web, old-school methods appear to still dominate more complex forms of participation in MCS. This has two main implications for the engagement of scuba divers in MCS. First, word of mouth and promotion of MCS through trustworthy organisations and the scientific community [5,8,23,29] should continue to be endorsed as successful methods of direct face-to-face engagement. Similarly, direct contact with participants throughout the scientific process and beyond (feedback, recognition) remains a key determinant of the positive experience of citizen scientists, particularly for demanding projects, and therefore needs to be retained above all [8,11–12,51]. Second, the internet clearly needs to be better exploited for the purpose of promoting MCS to potential participants [27,57,71]. The level of commitment to entice scuba divers through the internet should go beyond the good design and maintenance of websites showcasing MCS projects. The social networks and their structure offer important and multiple opportunities for promotion, particularly because scuba divers tend to make large use of them to discuss their sport and display images and videos of underwater environments and wildlife [4,57].

Scuba divers who participated in MCS did so in an active role of data collectors, with some prior training involved. While most divers collected data under the supervision of scientists, some participated in senior roles such as coordinator. Finally, a good proportion of the scuba divers were not limited to collecting data but were also involved in data handling and dissemination. These have been demonstrated to be activities particularly sought after by scuba diving citizen scientists [23]. The satisfactory elements of MCS participation relayed by the scuba divers seem to represent a perfect balance between contribution (by the participants to science) and education (by the scientists to the participants). However, participants acknowledged the financial challenges that MCS projects tend to face, and while they were satisfied with communication during participation, they lamented lack of feedback following participation [8,11,23].

While the results paint a positive picture of the scuba diver participating in MCS as a willing, active and inquisitive citizen scientist, there are some evident gaps making this image incomplete. On the one hand, participation did not include the use of any particular technology beside cameras and standard diving or monitoring equipment; this lack of new technology was lamented by the scuba divers. On the other hand, opportunistic MCS projects simply requiring the use of social media platforms to share data, particularly images, did not feature. Therefore, the picture presented here appears to be that of an experienced scuba diver who, while possessing all the qualities of a good citizen scientist, does not fully exploit the potential to explore the diversity of MCS available. It is likely that this potential is not exploited due to factors beyond the diver’s control, such as poor communication and marketing of certain MCS projects. In addition, the scuba diver depicted here does not receive the minimum stimulation and gratification required to feel “rewarded”. As it was evident that scuba divers participated in MCS for altruistic reasons, a direct acknowledegement (not necessarily public) of their efforts in the form of tangible feedback could provide a form of reward [8,23]. Several publications based on MCS acknowledge the contributions of scuba divers who participated and give them due credit for shared information and media (S1 Table). Scuba divers are likely to feel rewarded by such recognition; thus announcing it at the onset of and after an MCS project could be a simple step to satisfy the participants. Similarly, there are several opportunistic CS platforms in social media, enabling scuba divers to ask scientists for help with species identification or other matters. In such cases, citizen scientists can be seen as being indirectly rewarded by receiving an answer to their queries.

Providing access to new technologies to carry out tasks in the scientific process could be an additionally satisfying element for scuba divers, particularly those with more experience who tend to become more specialised and demanding of their sport [68–70]. This particular step could be decisive in re-engaging old citizen scientists and retaining present ones, and holds strong marketing potential for dive centres, which can sell courses and specialties (e.g. Diving Propulsion Vehicle or DPV, Peak Performance Buoyancy, photogrammetry) enabling clients to participate in more technologically advanced CS (e.g. requiring the use of DPV). While it is possible that opportunistic MCS is not stimulating enough for scuba divers, particularly experienced ones, it holds value as a type of MCS able to engage a broad spectrum of people with different backgrounds and experiences. It can represent a useful recruitment tool for potential citizen scientists [5,23]. The fact that it did not really feature in the data on participation suggests that greater promotion and engagement efforts need to be made to guarantee its future.

### Lessons from potential participants in MCS

The results of this study clearly show that participation in CS among scuba divers was relatively limited. Nevertheless, the majority of those divers who had never participated in CS declared to have been interested in MCS, particularly in those fields that tend to attract and involve scuba divers in MCS in the first place [3] (S1 Table). When explaining the reasons behind their interest, scuba divers provided answers echoing the recognised benefits of MCS and CS in general, as well as the intrinsic motivations normally pushing scuba divers to participate in MCS [22,24]. Additionally, scuba divers who had never participated in MCS, including those who showed interest and those who did not show interest in future participation, clearly felt that incentives like money and gifts would not be relevant in the decision whether or not to participate, which was instead dictated by other factors like time and knowledge of MCS. The scuba divers who had been interested in MCS showed an intention to participate in the future. Reasons why scuba divers had not been able to participate in the past tended to be partly beyond the divers’ control, as they were related to time and distance [5]. The scuba divers also felt that having limited knowledge of CS would dismiss any chance of participation. Finally, divers included the inaccessibility of MCS projects from a promotional point of view as a reason precluding their participation in MCS. More specifically, they lamented how no one had previously approached them to talk about MCS. The study gave an opportunity for potential participants in MCS to declare what actions would help them overcome any barrier to participation. Indeed, what divers desired was better promotion and engagement strategies, also based on direct contact with a person who would talk to them about CS, as well as better accessibility through dive centres and training opportunities.

The picture of the potential participant in MCS depicted here is very positive, in that there is a good disposition to participate in MCS, underpinned by altruistic motivations, and few barriers preventing participation [24]. However, it is clear that some actions need to be taken in order to eliminate these barriers, thus opening the horizon for much greater participation in MCS by scuba divers, with all its cascading benefits. Key role players in this scenario comprise those who manage MCS projects and those who should see a benefit in supporting MCS, including dive centres.

Behind most MCS projects is a single scientist or a group of scientists in a given field who, depending on the nature of a project, need to achieve a set of research and educational goals. Naturally, MCS projects would not see more than a few disciplines interacting, unless it is required by the scientific goals of projects. Consequently, MCS projects are likely to lack the input of other disciplines and skillsets, which may not be needed for the achievement of research goals, but would be critical in the processes of promotion or marketing, citizen engagement and communication throughout [8]. Limited funding in most MCS projects will make it the more difficult to consider involving and paying professionals to manage these processes. At any rate, MCS projects that wish to be successful and to achieve all set goals cannot avoid addressing this matter. As a minimum, scientists or other members coordinating MCS projects need to become acquainted with the art of promotional and marketing communication with the scuba diving community. This can happen easily and is already happening, for example in the context of large-scale interdisciplinary research projects, which demand the exchange of knowledge and skills among disciplines, as well as concerted efforts towards public engagement and scientific dissemination. Potential participants in MCS tend to feel that participation should require some level of scientific knowledge or skill, resulting in discouragement and determent [5,24,29]. Since a good proportion of MCS projects involving scuba divers only require basic diving skills and provide training, it is important to avoid any discouragement and misinterpretation of MCS. This should be achieved through large-scale information campaigns about the definition, nature and purpose of MCS across the circles frequented by scuba divers (e.g. certifying agencies, dive centres, dive clubs, social media groups and online diving magazines).

Dive centres play a central role in mediating the relationship between scuba divers and the marine environment. Not only do they represent commercial enterprises but also the hub of congregation of members in the scuba diving community, from dive schools and dive clubs to scientists and explorers. The importance of dive centres recognising the full marketing and economic potential that MCS represents for them has been stressed in recent research [7,9,32,43,57]. Dive centres ought to give support to MCS not simply for altruistic reasons, but especially for the benefit of their business. Examples of the tangible benefits for dive centres include the sale of a range of products such as courses, specialties, diving equipment, guide books, identification keys and slates, as well as the organisation of special events and exclusive dives at upmarket prices. At the dawn of a new sustainable diving industry era, the support for MCS should represent a natural step for dive centres, as it would result in the improvement of their image and the possibility to build their client-base, with economic benefits. Further, MCS support by dive centres and dive operators who can be appreciated for their experience and their local ecological knowledge, can result in benefits linked to managerial decisions, for example in marine reserves [9,43]. In line with requirements by specialised scuba divers to seek new and interesting experiences, dive centres can use MCS opportunities to satisfy these requirements and retain their existing clientele as well [12]. New MCS projects are looking into the possibility of engaging dive centres by offering benefits such as the restitution of collected data (by citizen scientists) in the form of a marketable product, such as three-dimensional maps of dive sites [72].

Scuba divers who had never been interested in MCS evidently possessed very little or no knowledge at all of CS in general. They agreed that their lack of interest was mainly due to this lack of knowledge [29]; those who had some understanding perceived MCS as an activity standing in the way of their enjoyment as scuba divers. It is acceptable that not all scuba divers can be involved in MCS, due to reasons beyond their control as well as reasons of noninterest. Nevertheless, some scuba divers were still open to hearing about MCS, provided that someone would give them such an opportunity [24]. Divers appeared to be satisfied with having someone simply coming to the dive centre to talk to them about CS, which suggests a certain degree of availability on behalf of the divers to establish a direct contact with CS.

Aside from direct contact with potential participants, using various channels to broadcast information on what CS is and what it can do, for example for society and to solve global issues, is generally a recommendation of many CS scholars [30]. Following this recommendation through the support of governments and scientists can help remove some of the basic prejudice that CS is either elitist or excessively task-loaded, time-consuming and cumbersome. It is true that some MCS projects require extra efforts compared with others [5]. However, potential citizen scientists who wish to put minimal effort into participation can still be included if the prejudice behind CS is eliminated. In particular, opportunistic projects inviting divers to share images they possess on social networks can be the kind attracting such divers, who can still enjoy their sport while also contributing to science, with no diversion from their plans and activities. Projects including passive participation, such as those monitoring the physiological parameters of divers and downloading data from dive computers, belong to a similar category. For these projects, as for any MCS project, the development of effective interfaces to present, educate and motivate potential participants is critical. The interface, for example a website, needs to also clearly display or foresee the relevant outcomes of participation, in order to project the scuba diver into what would be the tangible achievements of an MCS initiative [5,24]. Based on the declarations of scuba divers not interested in MCS, dive centres remain a key potential interlocutor and promoter of MCS, thus the benefits that MCS support can offer to dive centres should be considered seriously [7,32,57].

### Case-study-based differences in MCS participation and interest

This study shows a variability of responses by scuba divers both across case studies and based on the sampling method used. From the data, excluding responses from the online survey, it was evident that scuba divers interviewed in Europe were the most involved and the most interested in MCS, compared with scuba divers at the African case study. An interest in MCS and in future participation in MCS was also lowest among the scuba divers of Ponta do Ouro compared with their European counterparts. Scuba divers in Ponta do Ouro possessed the least diving experience in the study, and since diving experience tended to influence participation and interest in MCS, this could have played a partial role in determining the variation among case studies. In addition, Sub-Saharan countries including Mozambique and South Africa have featured infrequently in the panorama of published literature pertaining to MCS [3]. This issue is reflected in the limited knowledge that scuba divers in Ponta do Ouro claimed to have regarding CS in this study, and in documents such as the National Marine Research Plan for South Africa [73], which confirms how CS remains underrepresented in African countries. However, the recent advent of MCS projects in these areas, focusing on large-bodied marine fauna, signifies new possibilities for scuba divers of different experience levels to easily participate, given the minimum commitment for some of these projects (e.g. ID the Leopard Shark, organised by the Marine Mega fauna Foundation) and the charismatic nature of others [52,74].

The data from different case studies also highlight the prominence of certain types of MCS at different locations. For example, the Portofino case study was characterised by a strong interest in MCS focusing on medical and diving safety research. This reflects the current exposure of the local scuba diving tourism industry to research by DAN Europe, which also has a base in the area of Portofino. In addition, the Italian scuba diving tourism industry tends to have a strong safety culture compared with other European countries [75]. Paradoxically, DAN Europe has its headquarters in Malta, where the interest in medical and diving safety research was minimal among the divers. This result may be explained by factors such as familiarity to a diving destination (e.g. Portofino divers are loyal divers [43] and likely more knowledgeable of local CS initiatives) or time of sampling (summer in Italy vs. autumn in Malta). At any rate, the enormous potential offered by the experienced divers of Malta should be exploited across all fields of scuba diving MCS, particularly the ones which have well established roots at the location.

The diversity of MCS projects across space exemplifies the multifaceted and flexible qualities of CS, therefore should always be supported, particularly since many CS projects tend to be designed to address issues that are important to a given area and to raise public awareness of local problems. In addition, MCS projects working in the same areas should naturally join forces to enhance their exposure, promote CS and assist each other. For instance, Portofino is also known for hosting regular monitoring rounds organised by Reef Check Italia [43]. Therefore, it should be expected that scuba divers visiting the small area of Portofino have an understanding of the main CS initiatives available, of their common goals and of their support and respect for one another. Certain locations can also represent key spots for the monitoring of important changes in the seas and oceans, such as the introduction and spreading of alien and invasive species (S1 Table). Malta is an example of this, as it is placed in the centre of the southern Mediterranean Sea. Here, the invasion of alien species coming from the Red Sea to the east and the Atlantic Ocean to the west can be monitored and reported by diving citizen scientists using various techniques.

The results from the online survey clearly highlight the bias that can derive from certain methods of data collection, such as snowball and convenience sampling. These data, nevertheless, provided a good snapshot of those scuba divers who display a true passion for and connection with CS. The online survey also demonstrates how both active and potential citizen scientists can be easily engaged through the social networks and via email. Future research of this type should consider deploying parallel methods of sampling for comparative purposes, or to use snowball sampling in order to reach citizen scientists more effectively.

### Enhancing scuba diving MCS: A conceptual diagram

**Fig 6:**
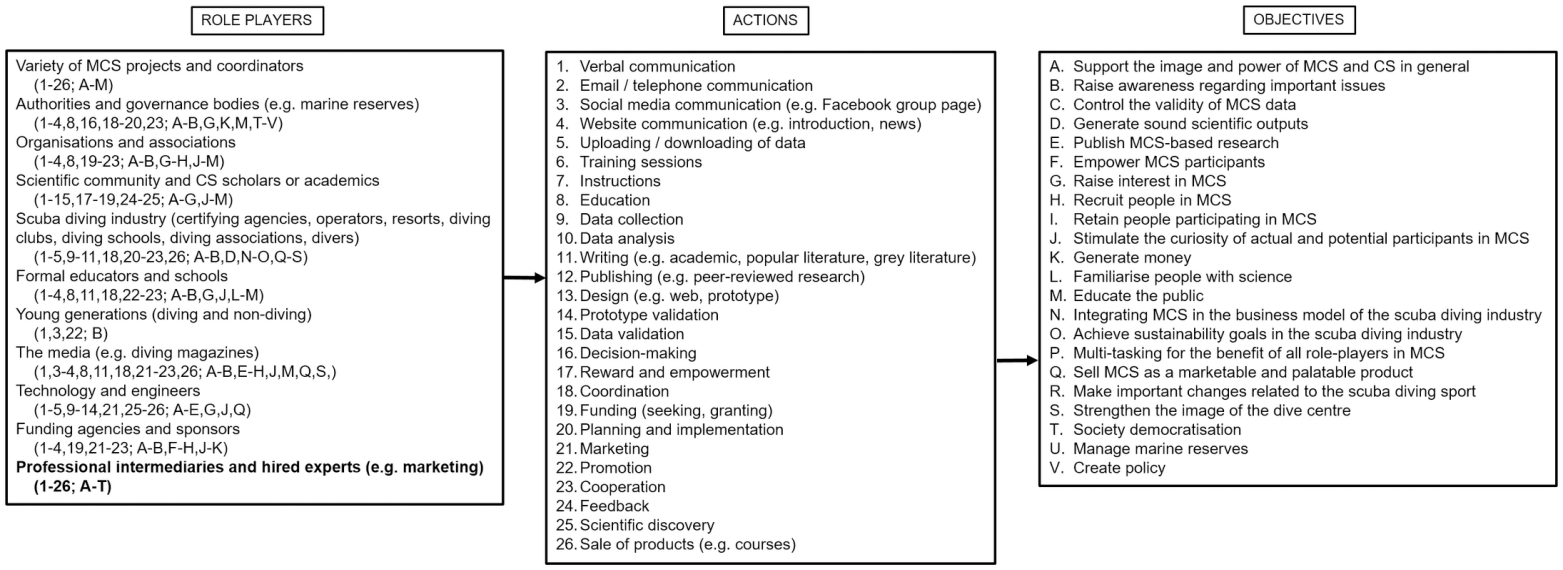
Conceptual diagram of role players, actions and objectives required for the success of MCS involving scuba divers. The arrows indicate how role players are intended to take one or more of the listed actions to achieve one or more of the outlined objectives. Numbers and letters in brackets indicate the actions and objectives for each role player, respectively. A number of actions and objectives overlap between role players, highlighting the integrated nature of MCS management.

From the results and the general recommendations of this study, a conceptual diagram was created, shown in Fig 6. The diagram lists groups of role players on the left-hand side, groups of actions in the middle, and groups of objectives required for successful MCS engaging scuba divers on the right-hand side. The boxes are connected by arrows, showing a unidirectional flow starting with the role players, who are required to take one or more of the actions listed in the second box to achieve one or more of the objectives outlined in the third box. From the diagram it is clear that there are different groups of role players who are crucial in maximising the potential of scuba diving MCS. Each group would be engaged in different actions depending on its role and objectives. For example, MCS projects and coordinators would be expected to take the most actions, ranging from project planning and funding application to promotion, communication, education, training, data validation, feedback, empowerment of participants and publishing. The main objectives of MCS projects and coordinators would comprise most of the objectives listed in Fig 6, such as raising awareness, recruiting participants, retaining participants, familiarising people with science, educating the public, and generating sound scientific outputs.

Role players such as governments and the broader scientific community would be expected to use various forms of communication and planning in order to support MCS and raise interest in and responsibility towards MCS. Obviously, a good part of the scuba diving industry, such as certifying agencies, diving associations, dive operators and divers, play an important role in supporting, marketing and implementing MCS into the scuba diving business model. The world of technology needs to work hand in hand with MCS by providing new platforms and prototypes that can be useful for the purpose of valid data collection, and attractive for the scuba diving industry. Finally, formal educators, including schools, have the task of introducing MCS to the younger generations through experimenting with the research process, in collaboration with scientists.

It is important to note that some role players are often unable to deliver objectives, due to task loading, financial constraints, lack of skills, or other factors. This issue, which has been extensively discussed in CS and MCS literature, calls for the introduction of central and multitasked roles in effective MCS. These roles would be able to meet the demands of the market and assist MCS projects with promotion, implementation and management. In Fig 6, these are referred to as professional intermediaries. While the world of scuba diving MCS may still be poorly acquainted with these roles, propositions have already been advanced and tested in more than a single occasion. An example is a recent elective course entitled “Citizen Science, natural heritage and recreational diving”, launched in Italy in 2017 as a joint collaboration between marine biologists, engineers and social scientists at academic institutions (Italy and South Africa), Reef Check Italia, PADI and Project AWARE, and DAN Europe [76]. The idea behind the course is to equip scuba diving marine biologists with a suite of skills capable of turning them into multitasked MCS coordinators, involved in aspects of MCS projects such as promotion and engagement. These people would ultimately be collocated as professionals at dive centres, taking a new role in the business model of dive centres. While the course is at its experimental phase, it is an attempt to meet the numerous demands of scuba diving MCS from public engagement and scientific education, to becoming a marketable product for the scuba diving industry. Another example would be that of hired experts (Fig 6), whose job would solely focus on the proper addressing of different aspects of MCS management, such as marketing or implementation. For instance, DAN Europe has been working on internship programmes designed to train future contracted experts, called Diving Safety Officers, whose job would involve the integration of safety CS at the dive centre, among other duties [77–78].

## Conclusions

CS is increasingly recognised and supported as a critical tool to strengthen the relationship between society and science through education and engagement, with win-win benefits. In particular, MCS is gaining momentum due to a growing interest of society in marine environments and marine issues. Scuba diving significantly increases the potential of MCS, thanks to the skills and behavioural properties of people who participate in the sport. However, scuba diving MCS faces challenges that are partly similar to those of other CS initiatives, and partly peculiar to the characteristics of scuba diving as a sport and a form of tourism. This paper sheds light on these challenges, by voicing the opinions of scuba divers who have actively participated in MCS and scuba divers who could participate in the future.

From the results, it is clear that scuba divers have a positive inclination towards MCS. However, retention of current volunteers and engagement of future participants in MCS may be made difficult by a lack of new stimuli in the case of the former group, and an overall lack of knowledge of CS in the case of the latter group. Evidently, the scuba diving industry, particularly dive centres, play a critical role in assisting CS initiatives, by making MCS publicly known. However, a proper integration of MCS into the scuba diving industry would necessitate the effort of professional intermediaries and hired experts working alongside the scientific community and the diving community. A good future for MCS sees the facilitation, by these new multitasked roles, in the promotion, implementation and coordination of MCS projects; the cooperation and co-support of geographically overlapping MCS projects; the broader engagement of scuba divers in MCS projects with different characteristics and demands; the accessibility of new technologies for more specialised scuba divers; the integration of MCS into the business model of dive centres; and the acquisition of new skills for MCS marketing and communication. Such facilitation, together with the continuing support of government, the scientific community and scuba diving industry, should guarantee the successful exploitation of scuba divers for MCS.

## Supporting information

S1 Table Published papers on MCS involving scuba divers from 2015 until 2018. The table aims to complement the review by Thiel et al., (2014) on MCS. The list of references was generated through a search in Google Scholar, Scopus, and Web of Science, using the key phrases “Marine Citizen Science” and “scuba diving”, “Citizen Science” and “scuba divers”, and “participatory research” and “scuba diving”. This appendix table does not include reviews discussing MCS.
(PDF)

S1 Questionnaire English version of the questionnaire survey used for this study. (PDF)

S2 Questionnaire Italian version of the questionnaire survey used for this study. (PDF)
