# Supplementary material for: Stirring the strategic direction of scuba diving marine Citizen Science: A survey of active and potential participants
Source: PLoS One. 2018 Aug 16;13(8):e0202484. doi: 10.1371/journal.pone.0202484 (PMC6095588; doi:10.1371/journal.pone.0202484)
Supplement: S1 Table — The table aims to complement the review by Thiel et al., (2014) on MCS. The list of references was generated through a search in Google Scholar, Scopus, and Web of Science, using the key phrases “Marine Citizen Science” and “scuba diving”, “Citizen Science” and “scuba divers”, and “participatory research” and “scuba diving”. This appendix table does not include reviews discussing MCS. (PDF) [file pone.0202484.s001.pdf]

S1 Table.

Title	Location	Field of research	Main method/contribution by scuba divers
Anderson LG, Chapman JK, Escontrela D, Gough CL. The role of conservation volunteers in the detection, monitoring and management of invasive alien lionfish. Manag Biol Invasion. 2017;8(4): 589-598.	Belize	Lionfish monitoring	Opportunistic and transect-based observation of species
Araujo G, Snow S, So CL, Labaja J, Murray R, Colucci A, et al. Population structure, residency patterns and movements of whale sharks in Southern Leyte, Philippines: results from dedicated photo-ID and citizen science. Aquat Conserv. 2017;27(1): 237-252.	Philippines	Whale shark identification and monitoring	Internet-based image sharing and information
Aylesworth L, Phoonsawat R, Suvanachai P, Vincent AC. Generating spatial data for marine conservation and management. Biodivers Conserv. 2017;26(2): 383-399.	Thailand	Species distribution of seahorses	Sightings information shared on database
Azzurro E, Bariche M. Local knowledge and awareness on the incipient lionfish invasion in the eastern Mediterranean Sea. Mar Freshw Res. 2017;68(10): 1950-1954.	Lebanon, Mediterranean Sea	Monitoring of lionfish invasions using local knowledge	Questionnaire survey (local ecological knowledge)
Balistreri P, Spiga A, Deidun A, Gueroun SK, Yahia MND. Further spread of the venomous jellyfish *Rhopilema nomadica* Galil, Spannier & Ferguson, 1990 (Rhizostomeae, Rhizostomatidae) in the western Mediterranean. BioInvasions Rec. 2017;6(1): 19-24.	Western Mediterranean Sea	Recording of alien jellyfish	Sighting of species
Bariche M, Azzurro E. Enhancing early detection through social networks: a Facebook experiment. 41st CIESM. 2016;41: 413.	Lebanon, Mediterranean Sea	Monitoring of invasive marine species	Images shared on social network (Facebook)
Boavida J, Assis J, Reed J, Serrão EA, Gonçalves JM. Comparison of small remotely operated vehicles and diver-operated video of circalittoral benthos. Hydrobiologia. 2016;766(1): 247-260.	Portugal	Benthic biodiversity assessment	Video recording along transects
Branchini S, Meschini M, Covi C, Piccinetti C, Zaccanti F, Goffredo S. Participating in a citizen science monitoring program: implications for environmental education. PloS One. 2015;10(7): e0131812.	Red Sea	Coral reef monitoring	Questionnaire survey (ecological information)
Branchini S, Pensa F, Neri P, Tonucci BM, Mattielli L, Collavo A, et al. Using a citizen science program to monitor coral reef biodiversity through space and time. Biodivers Conserv. 2015;24(2): 319-336.	Red Sea	Coral reef monitoring	Questionnaire survey (ecological information)
Burdeno D. Expanding the rescue a reef program: assessing efficacy of citizen scientists and adding massive species. 2016. [cited 15 January 2018]. In: Internship Reports 14 [Internet]. Available from: https://scholarlyrepository.miami.edu/rsmas_intern_reports/14.	Florida	Restoration of staghorn coral	Restoration outplants
Busch JA, Bardaji R, Ceccaroni L, Friedrichs A, Piera J, Simon C, et al. Citizen bio-optical observations from coast-and ocean and their compatibility with ocean colour satellite measurements. Remote Sens. 2016;8(11): 879.	Ireland; Spain	Monitoring of marine processes and water quality parameters using various technologies	Smartphone data collection using app, Secchi Disk, Revised Forel-Ule Scale, TrandiCam, and Do It Yourself kits
Carballo-Cárdenas EC, Tobi H. Citizen science regarding invasive lionfish in Dutch Caribbean	Dutch Caribbean	Drivers and barriers to	Questionnaire survey (willingness to

MPAs: drivers and barriers to participation. Ocean Coast Manage. 2016;133: 114-127.		participation in lionfish invasion control	detect species and remove species)
Castilla AM, Riera R, Humaid MA, Garland Jr T, Alkuwari A, Muzaffar S, et al. Contribution of citizen science to improve knowledge on marine biodiversity in the Gulf Region. J Assn Arab Univ Basic Appl Sci. 2017;24: 126-135.	Qatar and Gulf Region	Sea snake identification and distribution	Interviews (ecological information)
Cerrano C, Milanese M, Ponti M. Diving for science-science for diving: volunteer scuba divers support science and conservation in the Mediterranean Sea. Aquat Conserv. 2017;27(2): 303-323.	Italy, Mediterranean Sea	Environmental monitoring, monitoring of bottom habitats	Observation of presence/absence and abundance of taxa, environmental information from dive computers
Chatzigeorgiou G, Faulwetter S, Dailianis T, Smith VS, Koulouri P, Dounas C, et al. Testing the robustness of Citizen Science projects: evaluating the results of pilot project COMBER. Biodivers Data J. 2016;4: e10859.	Aegean Sea	Marine biodiversity monitoring	Observation of presence/absence and abundance of taxa
Cialoni D, Pieri M, Balestra C, Marroni A. Dive risk factors, gas bubble formation, and decompression illness in recreational SCUBA diving: analysis of DAN Europe DSL data base. Front Psychol. 2017;8: 1587. doi: 10.3389/fpsyg.2017.01587.	Europe	Retrospective analysis of physiological monitoring of scuba divers	Dive computer data and passive participation (ecodoppler)
Cigliano JA, Ballard HL. Citizen science for coastal and marine conservation. 2017. Abingdon: Routledge.	NA	Marine conservation	Mixed methods
Cigliano JA, Meyer R, Ballard HL, Freitag A, Phillips TB, Wasser A. Making marine and coastal citizen science matter. Ocean Coast Manage. 2015;115: 77-87.	Belize; California	Gastropod mollusk monitoring, marine protected area monitoring, conservation	Metrics measurement of species along transects, baselines for MPA monitoring, MPA monitoring
Clauson-Kaas S, Richardson K, Rahbek C, Holt BG. Species-specific environmental preferences associated with a hump-shaped diversity/temperature relationship across tropical marine fish assemblages. J Biogeogr. 2017;44(10): 2343-2353.	Caribbean	Fish species richness in relation to environmental parameters	Observation and identification of species
Couturier LI, Jaine FR, Kashiwagi T. First photographic records of the giant manta ray *Manta birostris* off eastern Australia. PeerJ. 2015;3: e742.	Eastern Australia	Manta ray identification and monitoring	Shared photographs and video recordings
Crocetta F, Gofas S, Salas C, Tringali LP, Zenetos A. Local ecological knowledge versus published literature: a review of non-indigenous Mollusca in Greek marine waters. Aquat Invasions. 2017;12(4): 415-434.	Greece	Non-indigenous mollusk inventory	Images shared online with additional information
D'Alelio D, Luongo G, Di Capua I. Plankton food for benthic fish: de visu evidence of trophic interaction between rainbow wrasse (*Coris julis*) and pelagic tunicates (*Pegea confoederata*). AIOL. 2017;8(2): 235-241.	Italy	Trophic interaction and monitoring of salps	Observation of species and photographic frames
Dailianis T, Akyol O, Babali N, Bariche M, Crocetta F, Gerovasileiou V, et al. New Mediterranean biodiversity records. Mediterr Mar Sci. 2016;17(2): 608-626.	Mediterranean Sea	New biodiversity records	Observations, shared photos, videos and specimens
Deidun A, Sciberras J, Sciberras A, Gauci A, Balistreri P, Salvatore A, et al. The first record of the white-spotted Australian jellyfish *Phyllorhiza punctata* von Lendenfeld, 1884 from Maltese waters (western Mediterranean) and from the Ionian coast of Italy. BioInvasions Rec. 2017;6(2): 119-124.	Malta; Ionian Sea, Italy	Recording of alien jellyfish species	Species sightings shared on online platform
Di Camillo CG, Ponti M, Bavestrello G, Krzelj M, Cerrano C. Building a baseline for habitat-	Central-Eastern	Mapping distribution of	Shared videos and additional information,

forming corals by a multi-source approach, including Web Ecological Knowledge. Biodivers Conserv. 2017;doi: 10.1007/s10531-017-1492-8.	Mediterranean Sea	habitat-forming corals	information from observation of presence/absence and abundance of taxa
Edgar GJ, Stuart-Smith RD, Cooper A, Jacques M, Valentine J. New opportunities for conservation of handfishes (Family Brachionichthyidae) and other inconspicuous and threatened marine species through citizen science. Biol Conserv. 2017;208: 174-182.	Australia	Handfish monitoring	Observation of species abundance and size along transects, photoquadrats
Forrester G, Baily P, Conetta D, Forrester L, Kintzing E, Jarecki L. Comparing monitoring data collected by volunteers and professionals shows that citizen scientists can detect long-term change on coral reefs. J Nat Conserv. 2015;24: 1-9.	British Virgin Islands	Coral reef monitoring	Observation of species abundance and cover along transects
Gerovasileiou V, Akel EHKh, Akyol O, Alongi G, Azevedo F, Babali N, et al. New Mediterranean Biodiversity Records (July, 2017). Mediterr Mar Sci 2017;18(2): 355-384.	Mediterranean Sea	New biodiversity records	Observations and shared photos
Gerovasileiou V, Chintiroglou C, Vafidis D, Koutsoubas D, Sini M, Dailianis T, et al. Census of biodiversity in marine caves of the Eastern Mediterranean Sea. Mediterr Mar Sci. 2015;16(1): 245-265.	Mediterranean Sea	Census of species in caves	Qualitative information through web for a and direct communications
Gerovasileiou V, Dailianis T, Panteri E, Michalakis N, Gatti G, Sini M, et al. CIGESMED for divers: Establishing a citizen science initiative for the mapping and monitoring of coralligenous assemblages in the Mediterranean Sea. Biodivers Data J. 2016;4: e8692.	Mediterranean Sea	Mapping and monitoring of coralligenous assemblages	Environmental data from dive computers and additional information, observation of species and their abundance, observation of threats, all uploaded onto online platform
Gerovasileiou V, Voultsiadou E, Issaris Y, Zenetos A. Alien biodiversity in Mediterranean marine caves. Mar Ecol. 2016;37(2): 239-256.	Mediterranean Sea	Information on alien species in caves	Information (ecological information) shared personally or through web
Giusti M, Cerrano C, Angiolillo M, Tunesi L, Canese S. An updated overview of the geographic and bathymetric distribution of *Savalia savaglia*. Mediterr Mar Sci. 2015;16(1): 128-135.	Mediterranean Sea	Gold coral mapping	Observation of species
Hesley D, Burdeno D, Drury C, Schopmeyer S, Lirman D. Citizen science benefits coral reef restoration activities. J Nat Conserv. 2017;40: 94-99.	Florida	Reef restoration	Restoration outplants
Hylton S, White WT, Chin A. The sharks and rays of the Solomon Islands: a synthesis of their biological diversity, values and conservation status. Pac Conserv Biol. 2018;23(4): 324-334.	Solomon Islands	Shark species checklist	Shared photographic or other evidence of species
Kienberger K, Prieto L. The jellyfish *Rhizostoma luteum* (Quoy & Gaimard, 1827): not such a rare species after all. Mar Biodivers. 2017;doi: 10.1007/s12526-017-0637-z.	Northeastern Atlantic Ocean and Alboran Sea	Historical distribution of jellyfish	Sightings of species, shared photos and videos through online platforms
Kittinger JN, Bambico TM, Minton D, Miller A, Mejia M, Kalei N, et al. Restoring ecosystems, restoring community: socioeconomic and cultural dimensions of a community-based coral reef restoration project. Reg Environ Change. 2016;16(2): 301-313.	O'ahu	Coral reef restoration	Interview (acceptance of restoration)
Lamine EB, Di Franco A, Romdhane MS, Francour P. Can citizen science contribute to fish assemblages monitoring in understudied areas? The case study of Tunisian marine protected areas. Estuar Coast Shelf S. 2018;200: 420-427.	Tunisia	Fish assemblages monitoring	Observation of presence/absence of species, observation of species density and size along transects
Loerzel JL, Goedeke TL, Dillard MK, Brown G. SCUBA divers above the waterline: using participatory mapping of coral reef conditions to inform reef management. Mar Policy.	US Virgin Islands	Mapping of reef habitats, marine protected area	Mapping of reef conditions, uses and threats using Google maps interface

2017;76: 79-89.		monitoring	
Loh TL, Tewfik A, Aylesworth L, Phoonsawat R. Species in wildlife trade: socio-economic factors influence seahorse relative abundance in Thailand. Biol Conserv. 2016;201: 301-308.	Thailand	Seahorse monitoring	Observation and metrics (sex, height, reproductive state) of species
Long SL, Azmi NA. Using photographic identification to monitor sea turtle populations at Perhentian Islands Marine Park in Malaysia. Herpetol Conserv Biol. 2017;12(2): 350-366.	Malaysia	Turtle monitoring using photos	Shared images online and via email, with additional information
Malpica-Cruz L, Chaves LC, Côté IM. Managing marine invasive species through public participation: Lionfish derbies as a case study. Mar Policy. 2016;74: 158-164.	Western Atlantic Ocean	Lionfish invasion control	Derbies
Mańko, M. K., A. Weydmann, and G. M. Mapstone. 2017. A shallow-living benthic Rhodaliid siphonophore: citizen science discovery from Papua New Guinea. Zootaxa. 2017;4324(1): 189-194.	Papua New Guinea	Siphonophores recording	Shared images via social network (Facebook)
Martin VY. Citizen Science as a means for increasing public engagement in science: presumption or possibility?. Sci Commun. 2017;39(2): 142-168.	Australia	Broad interest in MCS, marine species monitoring	Questionnaire survey (willingness to participate in sightings sharing)
Martin VY, Christidis L, Lloyd DJ, Pecl GT. Understanding drivers, barriers and information sources for public participation in marine citizen science. J Sci Commun. 2016;15(2): 1-19.	Australia	Marine species monitoring	Sightings of species shared through online platform
Martin VY, Christidis L, Pecl GT. Public interest in marine citizen science: is there potential for growth?. Bioscience. 2016;66(8): 683-692.	Australia	Broad interest in MCS, marine species monitoring	Questionnaire survey (willingness to participate in sightings sharing)
Martin VY, Smith L, Bowling A, Christidis L, Lloyd D, Pecl GT. Citizens as scientists: what influences public contributions to marine research?. Sci Commun. 2016;38(4): 495-522.	Australia	Broad interest in MCS, marine species monitoring	Questionnaire survey (willingness to participate in sightings sharing)
Mehrotra R, Scott CM. Species inventory of sea slugs (Gastropoda: Heterobranchia) for Koh Tao, Thailand, with 25 first records for Thai waters. Mar Biodivers. 2016;46(4): 761-771.	Thailand	Sea slug inventory using images	Images shared through social media or personal interviews, roving belt surveys
Meyers EK, Tuya F, Barker J, Jiménez Alvarado D, Castro-Hernández JJ, Haroun R, et al. Population structure, distribution and habitat use of the Critically Endangered Angelshark, *Squatina squatina*, in the Canary Islands. Aquat Conserv. 2017;27(6): 1133-1144.	Canary Islands, Spain	Angelshark identification and monitoring	Location and information (environmental and ecological) of encounters with species, shared through online platform
Mieras PA, Harvey-Clark C, Bear M, Hodgin G, Hodgin B. The economy of shark conservation in the Northeast Pacific: the role of ecotourism and Citizen Science. Adv Mar Biol. 2017;78: 121-153.	Northeast Pacific Ocean	Shark monitoring	Sighting and identification of species
Miyazaki Y, Murase A, Senou H. A natural history museum as a platform for accumulating verifiable information on non-native fishes: a Japanese example. Manag Biol Invasion. 2015;6(1): 105-110.	Japan	Alien species information through images and specimens	Shared images and specimens
Miyazaki Y, Murase A, Shiina M, Masui R, Senou H. Integrating and utilizing citizen biodiversity data on the Web for science: an example of a rare triggerfish hybrid image provided by a sport fisherman. J Coast Res. 2015;31(4): 1035-1039.	Japan	Biodiversity information through images shared online	Shared images
Morris DJ, Pinnegar JK, Maxwell DL, Dye SR, Fernand LJ, Flatman S, et al. Over 10 million seawater temperature records for the United Kingdom Continental Shelf between 1880 and 2014 from 17 Cefas (United Kingdom government) marine data systems. ESSD. 2018;10(1): 27-51.	UK	Seawater temperature monitoring	Data from dive computers
Mytilineou Ch, Akel EHKh, Babali N, Balistreri P, Bariche M, Boyaci YÖ, et al. New Mediterranean Biodiversity Records (November, 2016). Mediterr Mar Sci. 2016;17: 794-821.	Mediterranean Sea	New biodiversity records	Observations, shared photos, videos and specimens

Nazimi L, Robbins WD, Schilds A, Huveneers C. Comparison of industry-based data to monitor white shark cage-dive tourism. Tourism Manage. 2018;66: 263-273.	South Australia	Identification of sharks during cage diving	Shared images
Nimbs MJ, Larkin M, Davis TR, Harasti D, Willan RC, Smith SD. Southern range extensions for twelve heterobranch sea slugs (Gastropoda: Heterobranchia) on the eastern coast of Australia. Mar Biodivers Rec. 2016;9(1): 27.	Eastern Australia	Monitoring sea slugs geographic extension	Sightings and observation of species, shared images
Ormond R, Gore M, Bladon A, Dubock O, Kohler J, Millar C. Protecting Cayman Island sharks: monitoring, movement and motive. 69th GCFI. 2016;69: 14-27.	Cayman Islands	Shark monitoring	Observation of presence/absence of species and information (tags), shared online using platform
Özbek EÖ, Mavruk S, Saygu İ, Öztürk B. Lionfish distribution in the eastern Mediterranean coast of Turkey. J Black Sea/Medit Environ. 2017;23(1): 1-16.	Mediterranean coast of Turkey	Lionfish monitoring	Sightings reported on the social networks
Parkinson JE, Yang SY, Kawamura I, Byron G, Todd PA, Reimer JD. 2016. A citizen science approach to monitoring bleaching in the zoantharian *Palythoa tuberculosa*. PeerJ. 2016;4: e1815.	Japan	Monitoring bleaching of zoantharians	Colour assessment
Pikesley SK, Godley BJ, Latham H, Richardson PB, Robson LM, Solandt JL, et al. Pink sea fans (Eunicella verrucosa) as indicators of the spatial efficacy of Marine Protected Areas in southwest UK coastal waters. Mar Policy. 2016;64: 38-45.	Southwest UK	Monitoring of marine protected area using indicator species	Observation of abundance and metrics of species
Pittman SJ, Poti M, Jeffrey CF, Kracker LM, Mabrouk A. Decision support framework for the prioritization of coral reefs in the US Virgin Islands. Ecol Inform. 2017;doi: 10.1016/j.ecoinf.2017.09.008.	US Virgin Islands	Map-based decision-support tool using local ecological knowledge	Local ecological knowledge, participatory mapping tool in Google maps
Ragkousis M, Marmara D, Filiz H, Uyan U, Tuncer S, Romanidis-Kyriakidis G, et al. The northward expansion of *Synaptula reciprocans* (Echinodermata) in the Mediterranean Sea. J Black Sea/Medit Environ. 2017;23(3): 209-215.	Eastern Mediterranean Sea	Alien marine species monitoring	Images shared using online platform with additional information
Raoult V, David PA, Dupont SF, Mathewson CP, O'Neill SJ, Powell NN, et al. GoPros™ as an underwater photogrammetry tool for citizen science. PeerJ. 2016;4: e1960.	Great Barrier Reef, Australia	Coral reef habitat mapping using photogrammetry	GoPro photogrammetry
Roberts M, Hanley N, Williams S, Cresswell W. Terrestrial degradation impacts on coral reef health: evidence from the Caribbean. Ocean Coast Manage. 2017;149: 52-68.	Bonaire	Reef health monitoring	Observation of coral cover and visibility
Robertson DR, Simoes N, Gutierrez Rodriguez C, Pineros VJ, Perez-Espana H. An Indo-Pacific damselfish well established in the southern Gulf of Mexico: prospects for a wider, adverse invasion. JOSF. 2016;19: 1-17.	Gulf of Mexico	Alien species monitoring	Sighting of species
Roelfsema C, Thurstan R, Beger M, Dudgeon C, Loder J, Kovacs E, et al. A citizen science approach: a detailed ecological assessment of subtropical reefs at Point Lookout, Australia. PLoS One. 2016;11(10): e0163407.	South-East Queensland, Australia	Environmental monitoring, subtropical reef monitoring, environmental impact assessment	Mapping of habitat features, recording of substrate, fish and invertebrate composition, and quantifying impacts (e.g., occurrence of substrate damage, presence of litter)
Savage JM, Osborne PE, Hudson MD. Effectiveness of community and volunteer based coral reef monitoring in Cambodia. Aquat Conserv. 2017;27(2): 340-352.	Cambodia	Coral reef monitoring	Observation and count of indicator species along transects
Schultz JA, Cloutier RN, Côté IM. Evidence for a trophic cascade on rocky reefs following sea	British Columbia,	Trophic cascade	Species identification, observation of

star mass mortality in British Columbia. PeerJ 2016;4: e1980.	Canada	monitoring following sea star mass mortality	presence/absence and abundance
Sini M, Katsanevakis S, Koukourouvli N, Gerovasileiou V, Dailianis T, Buhl-Mortensen L, et al. Assembling ecological pieces to reconstruct the conservation puzzle of the Aegean Sea. Front Mar Sci. 2017;4: 347.	Aegean Sea	Information to map ecological features	Questionnaire survey (ecological knowledge)
Stuart-Smith RD, Edgar GJ, Barrett NS, Bates AE, Baker SC, Bax NJ, et al. Assessing national biodiversity trends for rocky and coral reefs through the integration of citizen science and scientific monitoring programs. Bioscience. 2017;67(2): 134-146.	Australia	Reef monitoring	Observation and count of species along transects, photoquadrats
Tolimieri N, Holmes EE, Williams GD, Pacunski R, Lowry D. Population assessment using multivariate time-series analysis: a case study of rockfishes in Puget Sound. Ecol Evol. 2017;7(8): 2846-2860.	Puget Sound, USA	Rockfish population assessment	Observation of abundance
Turicchia E, Cerrano C, Abbiati M, Ponti M. From Citizen Sciences to environmental quality assessment: the Portofino MPA case study. 41st CIESM. 2016;41: 552.	Italy, Mediterranean Sea	Sensitivity of assemblages in marine protected area	Observation of presence/absence and abundance of taxa
Van Moorsel GW, Bennema FP, Nijland R. First records of the sponge crab *Dromia personata* (Brachyura) in the Netherlands and its historical findings in the North Sea. Mar Biodiovers Rec. 2017;10(1): 28.	Netherlands	Recording of sponge crab	Sightings
Williams JL, Pierce SJ, Fuentes MM, Hamann M. Effectiveness of recreational divers for monitoring sea turtle populations. Endanger Species Res. 2015;26(3): 209-219.	Mozambique	Sea turtle monitoring	Sightings log, survey reporting sightings
Williams JL, Pierce SJ, Rohner CA, Fuentes MM, Hamann M. Spatial distribution and residency of green and loggerhead sea turtles using coastal reef habitats in southern Mozambique. Front Mar Sci. 2017;3: 288.	Mozambique	Sea turtle identification and distribution	Sightings log, photo-ID collection
Wright S, Hull T, Sivyer DB, Pearce D, Pinnegar JK, Sayer MD, et al. SCUBA divers as oceanographic samplers: the potential of dive computers to augment aquatic temperature monitoring. Sci Rep. 2016;6: 30164.	UK	Seawater temperature monitoring	Data from dive computers
