# Supplementary material for: Stirring the strategic direction of scuba diving marine Citizen Science: A survey of active and potential participants
Source: PLoS One. 2018 Aug 16;13(8):e0202484. doi: 10.1371/journal.pone.0202484 (PMC6095588; doi:10.1371/journal.pone.0202484)
Supplement: S1 Questionnaire — (PDF) [file pone.0202484.s002.pdf]

CITIZEN SCIENCE QUESTIONNAIRE 2015
SECTION A: DEMOGRAPHIC DETAILS

1. Gender?

M	1
F	2

2. Year of birth: ________

3. Education:

No school	1
Matric	2
Diploma or professional	3
Graduate	4
Master degree	5
PhD or equivalent	6

3.1. For answers 3 to 6 can you specify your main subject?

____

4. Which country are you from?

____

5. Marital status:

Single	1
Married	2
De facto	3
Divorced	4
Widow/er	5

6. Occupation:

Student	1
Paid work	2
Unpaid work	3
Unemployed	4
Retired	5

6.1. If you are/have been employed, what is/was your profession?

____

7. You grew up:

By the sea	1
Inland	2

SECTION B: DIVING EXPERIENCE

1. How many certifications do you hold in each of the following:

LEVEL	NUMBER
A. Basic (Open, Advanced, Rescue)	
B. Pro (Dive Master, Assistant Instructor/Instructor of any level and speciality)	
C. Speciality (including caves/caverns, ice, recreational Nitrox, but excluding all those under "technical" and "dry" categories below)	
D. Technical (all types/levels for Trimix, Rebreather, Side Mount, Deco Dive, DPV, etc.)	
E. Dry (Oxygen provider, Gas blender, Equipment specialist, Frist Aid, Boat operator, etc.)	
F. Anything that cannot be listed in the above or are unsure where to place, place here:	

2. Which certifying agency, among those that issued your certifications, represents you the most?

____

3. In what year did you start diving?

____

4. How many dives have you logged since you started diving?

____

5. How many dives a year do you log?

____

6. Is scuba diving your primary job?

YES	NO

SECTION C: CITIZEN SCIENCE

Citizen Science is defined as any involvement of volunteer participants in the generation of scientific knowledge, that is, observation, sampling, collection of data, and analysis of data, which enter the normal scientific production cycle, ideally leading to reports supporting management decisions and scientific publications.

1. As a diver, are you or have you been involved in Citizen Science?

YES	**If your answer was YES, please complete the rest of this section**
NO	**If your answer was NO, please move to section D**

2. How many Citizen Science projects have you participated in, including current ones?

2.1. Name of project:	2.2. No. times participated:	2.3. How long:

3. Please provide the following information about the latest Citizen Science project you are/have been involved in as a diver (tick boxes where appropriate):

3.1. Name of project: ____________ 3.2. Location: ____________ 3.3 How long have you participated in this CS project? ____________

3.4. Subject:

Biology, ecology and conservation	1
Medicine and safety	2
Technology and engineering	3
Social science	4
Other (specify):	5

3.5. Your role:

Training scientist	1
Coordinator	2
Active volunteer	3
Other (specify):	4

3.6. Type of data you collect (tick all that apply):

Images/video	1
Recordings from tool	2
Verbal communications	3
Written numbers/notes	4
Samples	5
I did not collect any data	6
Other (specify):	7

3.7. The data are (tick all that apply):

Environmental (e.g. water temperature)	1
Biological/ecological (flora)	2
Biological/ecological (fauna)	3
Physiological (myself or others)	4
Human behaviour (mine or others')	5
Social/cultural	6
I did not collect any data	7
Other (specify):	8

3.8. Place of data collection:

Beach/rocky shore	1
Shallow water	2
Deep water	3
I did not collect any data	4
Other (specify):	5

3.9. Geographic scale of project:

Local	1
Regional	2
National	3
International	4

3.10. Temporal span of project :

Once off	1
A few days	2
Weeks	3
Months	4
Years	5
Decades	6

3.11. Other duties aside from data collection (tick all that apply):

None	1
Maintenance (e.g. of equipment)	2
Tagging	3
Data entry	4
Data analysis	5
Reporting verbally or through email, web portal	6
Writing	7
Publishing	8
Other (specify):	9

3.12. No. volunteers involved in project:

A few tens or less	1
Hundreds	2
Thousands	3
Millions	4
Don't know	5

3.13. Your commitment to the project:

Once only	1
Once a year or so	2
Once a month or so	3
Once a week or so	4
More than once a week	5

3.14. Did you go through a selection process to participate?

YES	NO

3.15. Generally you collected data:

Alone	1
With other volunteers	2
With scientist	3
With scientists and volunteers	4
I did not collect any data	5

3.16. Compensation:

None	1
A gift	2
Money payment	3
Employment	4
Other (specify):	5

3.17. Did you receive training before participating?

No	1
Brief instructions	2
Basic training	3
Special training	4

3.18. Where have you heard about this project?

Organized group	1
Word of mouth	2
Web	3
Other (specify):	4

3.19. Materials generally provided:	By me	By the project	By local operators
Data sheets	1	1	1
Quadrat/tape measure	2	2	2
Diving equipment	3	3	3
Boats/watercraft	4	4	4
Infrastructure	5	5	5
Computer/tablet	6	6	6
Camera	7	7	7
Smartphone	8	8	8
GPS	9	9	9
Internet connection	10	10	10
Software and applications	11	11	11
Other (specify):	12	12	12

3.20. How far do/did you normally travel to partake in this CS project?

____

3.21. How much of your money do/did you normally spend for each trip dedicated to this CS project?

____

4. Please indicate your level of satisfaction with your experience as a Citizen Scientist in general:

	Unsatisfied	Neutral	Satisfied	Not applicable
Education received	1	2	3	4
Educational material	1	2	3	4
Training received	1	2	3	4
Equipment and tools provided	1	2	3	4
Quality of the data produced	1	2	3	4
Guidance by the coordinators	1	2	3	4
General preparation of volunteers	1	2	3	4
Overall contribution made by the volunteers	1	2	3	4
Networking and meeting people	1	2	3	4
The availability of new technology	1	2	3	4
Use of available technology	1	2	3	4
The funding the project receives	1	2	3	4
Project's webpage/social media	1	2	3	4
Overall outreach of the project	1	2	3	4
Communication throughout	1	2	3	4
Feedback on the results	1	2	3	4
Overall quality of the experience	1	2	3	4
Overall success of the project	1	2	3	4
The reward received	1	2	3	4
The contribution of the project to science	1	2	3	4
Other (specify):	1	2	3	4

5. Please indicate the reasons why you partook/partake in Citizen Science as a diver:

	Disagree	Neutral	Agree
For the sake of science	1	2	3
For my interest in the subject under study	1	2	3
For the sake of society/the environment	1	2	3
For medical and safety reasons	1	2	3
For the contribution I can give	1	2	3
For emotional satisfaction	1	2	3
For the knowledge gained	1	2	3
For the reward	1	2	3
For personal satisfaction	1	2	3
For public recognition	1	2	3
For networks and collaborations	1	2	3
To access new technology	1	2	3
For the sake of future generations	1	2	3
To spend time with like minded people	1	2	3
To acquire skills	1	2	3
To spend quality time with my family/friends	1	2	3
Other (specify):	1	2	3

6. As a diver, are you going to participate in Citizen Science in the future?

YES | NO

6.1. If your answer was NO, can you briefly explain why?

____

____

____

____

7. Are there any comments you wish to make regarding Citizen Science or the specific CS project you reported about?

____

____

____

____

____

Thank you! © Green Bubbles 2015 www.greenbubbles.eu

Green Bubbles

The Green Bubbles project has received funding from the European Union's Horizon 2020 research and innovation programme under the Marie Sklodowska-Curie grant agreement No 643712.
This document reflects only the authors' view. The Research Executive Agency is not responsible for any use that may be made of the information it contains.

SECTION D: WILLINGNESS TO PARTICIPATE IN CITIZEN SCIENCE

1. As a diver, have you ever been interested in Citizen Science?

YES	**Please answer questions 1.1. and 1.2.**

NO	**Please answer question 1.3.**

1.1. What type of Citizen Science interests you?	
Biology, ecology and conservation	1
Medicine	2
Technology and engineering	3
Social science	4
Other (specify):	5

1.2. Why is Citizen Science of interest to you?	Disagree	Neutral	Agree
I have a general interest in science	1	2	3
It deals with fields I am interested in	1	2	3
It makes a contribution to society and the environment	1	2	3
It makes a contribution to science	1	2	3
It is a powerful scientific tool	1	2	3
It engages citizens in developing an interest in science	1	2	3
It empowers people	1	2	3
It is educational	1	2	3
It incentivises citizens	1	2	3
it gives people an opportunity to make connections	1	2	3
it grants people access to new technology	1	2	3
It is a socialising experience	1	2	3
Others told me it is good	1	2	3
Other (specify):	1	2	3

1.3. Why is Citizen Science not attractive to you?	Disagree	Neutral	Agree
I do not really know much about it	1	2	3
I have no interest in science	1	2	3
It does not cover those fields I am interested in	1	2	3
It makes no contribution to science	1	2	3
It makes no contribution to society and the environment	1	2	3
It is not an effective scientific tool	1	2	3
It is a waste of time	1	2	3
It costs money	1	2	3
It exploits citizens as volunteers	1	2	3
It is not rewarding	1	2	3
It is not well organised	1	2	3
Others told me it is no good	1	2	3
It makes no good use of available technology	1	2	3
It is discriminatory towards people that have no skills	1	2	3
It is boring	1	2	3
It seems difficult	1	2	3
If I dive I just want to enjoy my time	1	2	3
I do not trust those who organise CS projects	1	2	3
I had a bad experience with CS before	1	2	3
Other (specify):	1	2	3

2. As a diver, would you be interested in participating in Citizen Science?

YES	**Please answer questions 2.1. and 2.2.**

NO	**Please answer question 2.3. and 2.4.**

2.1. Why have you not participated before?	Disagree	Neutral	Agree
I have/had no time	1	2	3
I am/was too young	1	2	3
I have/had limited knowledge of Citizen Science	1	2	3
I have/had problems with access (e.g. no car)	1	2	3
I have never been invited by anyone	1	2	3
I am unsure how to look for Citizen Science projects	1	2	3
I am unsure how to join a Citizen Science project	1	2	3
Available projects are usually far from where I live	1	2	3
There is/was no availability	1	2	3
I feel that I do not have the skills	1	2	3
Others tell/told me it is no good	1	2	3
It seems/seemed complicated	1	2	3
The technology was old (e.g. no use of smartphone)	1	2	3
I had no money to invest in it	1	2	3
My equipment is inadequate	1	2	3
The website of the project I like is unattractive	1	2	3
There was no training available	1	2	3
The operator did not give me a chance to volunteer	1	2	3
The provided material was poor and not in my language	1	2	3
Other (specify):	1	2	3

2.2. What would convince you to participate?	Disagree	Neutral	Agree
I am already convinced	1	2	3
Someone to come and talk about it	1	2	3
Logistic help (e.g. car lift)	1	2	3
An incentive like money	1	2	3
An incentive like a gift voucher or other	1	2	3
A training course	1	2	3
Promotional material in my language	1	2	3
Better media coverage	1	2	3
More availability	1	2	3
Better organisation	1	2	3
Better/new technology	1	2	3
If the operator gives me a chance to participate	1	2	3
A better website for the project	1	2	3
If I am provided with equipment to work with	1	2	3
Earning a higher income	1	2	3
Other (specify):	1	2	3

2.3. Why would you not be interested?	Disagree	Neutral	Agree
For the same reasons I agreed to in question 1.3.	1	2	3
I have no time	1	2	3
I am too young/too old	1	2	3
I have problems with access (e.g. no car)	1	2	3
It seems difficult	1	2	3
Available projects are far from where I live	1	2	3
I feel that I do not have the skills	1	2	3
There is no benefit to me	1	2	3
I earn no/too little money	1	2	3
Other (specify):	1	2	3

2.4. What would convince you to become interested?	Disagree	Neutral	Agree
Nothing	1	2	3
Someone to come and talk about it	1	2	3
Logistic help (e.g. car lift)	1	2	3
An incentive like money	1	2	3
An incentive like a gift voucher or other	1	2	3
Promotional material in my language	1	2	3
Better media coverage	1	2	3
More availability	1	2	3
Better organisation	1	2	3
Better/new technology	1	2	3
A nice website	1	2	3
A training course	1	2	3
If the operator invites me to participate	1	2	3
To be offered equipment to work with	1	2	3
Earning a higher income	1	2	3
Other (specify):	1	2	3

3. Are there any comments you wish to make regarding Citizen Science?

____

____

____

____

____

Thank you! © Green Bubbles 2015 www.greenbubbles.eu

Green Bubbles

The Green Bubbles project has received funding from the European Union's Horizon 2020 research and innovation programme under the Marie Sklodowska-Curie grant agreement No 643712. This document reflects only the authors' view. The Research Executive Agency is not responsible for any use that may be made of the information it contains.
