# Supplementary material for: Stirring the strategic direction of scuba diving marine Citizen Science: A survey of active and potential participants
Source: PLoS One. 2018 Aug 16;13(8):e0202484. doi: 10.1371/journal.pone.0202484 (PMC6095588; doi:10.1371/journal.pone.0202484)
Supplement: S2 Questionnaire — (PDF) [file pone.0202484.s003.pdf]

QUESTIONARIO SULLA CITIZEN SCIENCE 2015
SEZIONE A: DATI DEMOGRAFICI

1. Sesso?

M	1
F	2

2. Anno di nascita: ____________

3. Livello di educazione:

Niente scuola	1
Licenza elementare	2
Licenza scuola media inferiore	3
Diploma scuola media superiore o equivalente	4
Laurea (e.g. triennale, specialistica)	5
Educazione post laurea (e.g. dottorato)	6
Altro (specifica):	7

____

3.1. Se hai risposto da "4" a "7" alla domanda 3, qual è o è stata la tua materia principale?

____

4. Nazione di origine:

____

5. Stato civile:

Celibe/nubile	1
Sposato/a	2
Convivente	3
Divorziato/a	4
Vedovo/a	5

6. Occupazione:

Studente/ssa	1
Lavoro retribuito	2
Posiz. lavorativa non retribuita	3
Nessun impiego	4
Pensionato/a	5

6.1. Se sei o sei stato/a impiegato/a, qual è o è stata la tua professione?

____

7. Sei cresciuto/a:

Sul mare	1
Lontano dal mare	2

SEZIONE B: ESPERIENZA SUBACQUEA

1. Quante certificazioni possiedi per ciascuna delle seguenti categorie:

	Numero
a. Base (Open, Avanzato, Rescue/Salvamento)	
b. Pro (Dive Master, Assistente Istruttore/Istruttore di qualsiasi livello o specialità)	
c. Specialità (incluse grotte/caverne, ghiaccio, Nitrox ricreativo, ma escluse tutte le certificazioni sotto "tecniche" e "asciutte")	
d. Tecniche (tutti i tipi/livelli per Trimix, Rebreather, Side Mount, Deco Dive/Decompression, DPV, etc.)	
e. Asciutte (Somministrazione ossigeno, Gas Blender, Equipment, Primo Soccorso, Boat Operator, etc.)	
f. Per tutto ciò che non è riportato qui sopra o di cui non sei sicuro/a, aggiungi qui:	

2. Con quale didattica, tra quelle che hanno rilasciato le tue certificazioni, ti identifichi maggiormente?

____

3. In che anno hai iniziato ad immergerti? ____________

4. Numero totale di immersioni ad oggi? ____________

5. Quante immersioni fai all'anno in media? ____________

6. Lavori nell'industria subacquea?

Si	No

SEZIONE C: CITIZEN SCIENCE

COSA È LA CITIZEN SCIENCE?

La Citizen Science coinvolge volontari (non specializzati) in attivitá che prevedono la generazione di dati, (con osservazioni o campionamento) e/o l'analisi di questi stessi dati, a scopo scientifico.

1. Sei mai stato coinvolto in progetti di Citizen Science come subacqueo/a?

SI	Se hai risposto **SI**, completa il resto della **sezione C**
NO	Se hai risposto **NO**, vai alla **sezione D**

2. A quanti progetti di Citizen Science hai partecipato come sub?

2.1. Nome del progetto:

____

2.2. Quante volte hai partecipato:

____

2.3. Durata partecipazione:

____

3. Informazioni sull'ultimo progetto Citizen Science a cui hai partecipato come subacqueo/a:

3.1. Nome del progetto:

____

3.2. Luogo di svolgimento:

____

3.3. Durata della partecipazione:

____

3.4. Hai passato una selezione per partecipare?

____

3.5. Tema principale:

Biologia, ecologia, conservazione	1
Medicina e sicurezza	2
Tecnologia e ingegneria	3
Science Sociali	4
Altro (specifica):	5

____

3.6. Il tuo ruolo:

Ricercatore istruttore	1
Coordinatore	2
Volontario attivo	3
Altro (specifica):	4

____

3.7. Modalità di raccolta dati:

Immagini/video	1
Registrazioni audio	2
Comunicazioni verbali	3
Raccolta di numeri o appunti (dati)	4
Raccolta di campioni	5
Nessuna	6
Altro (specifica):	7

____

3.8. Tipo di dati raccolti:

Ambientali (es. temperatura dell'acqua)	1
Biologici/ecologici (flora)	2
Biologici/ecologici (fauna)	3
Fisiologici (tuoi o di altri)	4
Sul comportamento umano (tuoi o di altri)	5
Sociali/culturali	6
Nessuno	7

Altro (specifica):

____

3.9. Luogo di raccolta dati:

Spiaggia/scogliera	1
Acque basse/bassi fondali	2
Acque profonde/fondali profondi	3
Nessuno	4
Altro (specifica):	5

____

3.10. Scala geografica del progetto:

Locale	1
Regionale	2
Nazionale	3
Internazionale	4

3.11. Durata totale del progetto :

Un giorno	1
Pochi giorni	2
Settimane	3
Mesi	4
Anni	5
Decenni	6

3.12. Responsabilità al di fuori della raccolta dati:

Nessuna	1
Manutenzione (es. attrezzatura)	2
Tagging/Marcatura	3
Immissione dati (informatizzazione o in un registro)	4
Analisi dati	5
Riportare informazioni via email o simile	6
Scrittura (es. report)	7
Pubblicazioni	8
Altro (specifica):	9

____

3.13. Numero di volontari coinvolti:

Decine	1
Centinaia	2
Migliaia	3
Milioni	4
Non lo so	5

3.14. Il tuo impegno per il progetto:

Una sola volta	1
Una volta all'anno	2
Una volta al mese	3
Una volta a settimana	4
Più di una volta a settimana	5

3.15. In generale, hai raccolto dati:

Da solo	1
Con altri volontari	2
Con uno scienziato	3
Con scienziati e volontari	4
Non ho raccolto dati	5

3.16. Compenso:

Nessuno	1
Un regalo	2
Soldi	3
Impiego	4
Altro (specifica):	5

____

3.17. Preparazione

Nessuna	1
Briefing	2
Base	3
Specializzata	4

3.18. Come sei venuto a conoscenza del progetto?

Organizzatori	1
Passaparola	2
Internet	3
Altro (specifica):	4

____

3.19. Materiali forniti:

	Da me	Dal progetto	Da diving/scuola sub
Scheda raccolta dati	1	2	3
Quadrato/metro	1	2	3
Attrezzatura sub	1	2	3
Barca, gommone	1	2	3
Infrastruttura (es.laboratorio)	1	2	3
Computer/tablet	1	2	3
Macchina fotografica	1	2	3
Smartphone	1	2	3
GPS	1	2	3
Internet	1	2	3
Software	1	2	3
Altro (specifica):	1	2	3

____

3.20. Quanti chilometri hai viaggiato ogni volta che hai partecipato?

____

3.21. Quanti dei tuoi soldi hai speso ogni volta che hai partecipato?

____

4. Indica il tuo livello di soddisfazione per la tua esperienza di Citizen Science in generale:

	Insoddisfatto	Neutro	Soddisfatto	Non applicabile
a. Educazione ricevuta	1	2	3	4
b. Materiale di studio	1	2	3	4
c. Preparazione ricevuta	1	2	3	4
d. Attrezzature fornitemi	1	2	3	4
e. Qualità dei dati raccolti	1	2	3	4
f. Guida dei coordinatori	1	2	3	4
g. Preparazione generale dei volontari	1	2	3	4
h. Il contributo complessivo dei volontari	1	2	3	4
i. Nuove conoscenze e contatti	1	2	3	4
j. La disponibilità di nuove tecnologie	1	2	3	4
k. L'uso delle tecnologie disponibili	1	2	3	4
l. Fondi/finanziamenti disponibili	1	2	3	4
m.Promozione tramite internet	1	2	3	4
n. Attività di comunicazione in generale	1	2	3	4
o. Comunicazioni ricevute durante le attività	1	2	3	4
p. Comunicazione dei risultati	1	2	3	4
q. Qualità dell'esperienza	1	2	3	4
r. Successo generale	1	2	3	4
s. Premi ed incentivi ricevuti	1	2	3	4
t. Contributo alla scienza	1	2	3	4
u. Altro (specifica):	1	2	3	4

5. Indica le ragioni per cui hai partecipato, come subacqueo/a, nella Citizen Science:

	Non d'accordo	Neutro	D'accordo
a. Per la scienza	1	2	3
b. Perchè mi interessava l'argomento	1	2	3
c. Per la società e l'ambiente	1	2	3
d. Per il bene della medicina e della sicurezza	1	2	3
e. Per dare il mio contributo	1	2	3
f. Per la mia soddisfazione emotiva	1	2	3
g. Per aumentare il mio livello di conoscenza	1	2	3
h. Per il premio/incentivo	1	2	3
i. Per la mia soddisfazione personale	1	2	3
j. Per il riconoscimento pubblico	1	2	3
k. Per le collaborazioni e i contatti	1	2	3
l. Per accedere a nuove tecnologie	1	2	3
m. Per le generazioni future	1	2	3
n. Per passare tempo con persone come me	1	2	3
o. Per acquisire nuove competenze	1	2	3
p. Per stare con la famiglia e gli amici	1	2	3
q. Altro (specifica):	1	2	3

6. Hai intenzione di partecipare in futuro, come subacqueo/a, in attivitá di Citizen Science?

Si	No

6.1. Se la risposta è NO, puoi spiegarne il motivo?

____
____
____
____
____

7. Ci sono commenti che vuoi aggiungere sulla Citizen Science?

____
____
____
____
____

SEZIONE D: ATTITUDINI VERSO LA CITIZEN SCIENCE

COSA È LA CITIZEN SCIENCE?

La Citizen Science coinvolge volontari (non specializzati) in attivitá che prevedono la generazione di dati, (con osservazioni o campionamento) e/o l'analisi di questi stessi dati, a scopo scientifico.

1. Sei mai stato interessato, come subacqueo/a, alla Citizen Science?

SI	**Passa** alle domande **1.1**. and **1.2**.
NO	**Passa** alla domanda **1.3.**

1.1. Quale tipo di Citizen Science ti interessa?

Biologia, ecologia, conservazione	1
Medicina e sicurezza	2
Tecnologia e ingegneria	3
Scienze sociali	4
Altro (specifica):	5

____

	Non d'accordo	Neutro	D'accordo
**1.2. Perchè ti interessa la Citizen Science?**			
Mi interessa la scienza in generale	1	2	3
Ha a che fare con gli argomenti che mi interessano	1	2	3
Contribuisce al bene della società e dell'ambiente	1	2	3
Contribuisce alla scienza	1	2	3
È un potente strumento scientifico	1	2	3
Avvicina i cittadini alla scienza	1	2	3
Fa crescere le persone	1	2	3
È educativa	1	2	3
Incentiva i cittadini	1	2	3
Crea opportunità per stabilire nuovi contatti	1	2	3
Dà accesso a nuove tecnologie	1	2	3
È un'occasione per socializzare	1	2	3
Mi hanno detto che ne vale la pena	1	2	3
Altro (specifica):	1	2	3
**1.3. Perchè non ti interessa la Citizen Science?**			
Non ne so molto	1	2	3
Non ho interesse nella scienza	1	2	3
Non trattra gli argomenti che mi interessano	1	2	3
Non contribuisce alla scienza	1	2	3
Non contribuisce al bene della società e dell'ambiente	1	2	3
Non è uno strumento scientifico efficiente	1	2	3
È una perdita di tempo	1	2	3
È costosa	1	2	3
Sfrutta i cittadini come volontari	1	2	3
Non è gratificante	1	2	3
Non è organizzata bene	1	2	3
Altri mi hanno detto che non ne vale la pena	1	2	3
Non fa buon uso della tecnologia disponibile	1	2	3
È discriminatoria verso chi non ha le competenze	1	2	3
È noiosa	1	2	3
Sembra difficile	1	2	3
Se mi immergo lo faccio solo per divertirmi	1	2	3

Non mi fido degli organizzatori	1	2	3
Ho avuto una brutta esperienza a riguardo in passato	1	2	3
Altro (specifica):	1	2	3

2. Saresti interessato, come subacqueo/a, a partecipare in attività di Citizen Science?

SI	**Passa** alle domande **2.1**. and **2.2**.
NO	**Passa** alle domande **2.3**. and **2.4.**

	Non d'accordo	Neutro	D'accordo
**2.1. Perchè non hai mai partecipato prima?**			
Problemi di tempo	1	2	3
Troppo giovane	1	2	3
Conoscenza limitata della Citizen Science	1	2	3
Problemi logistici (e.g. trasporto)	1	2	3
Non mi é mai stato proposto	1	2	3
Difficile trovare iniziative di Citizen Science	1	2	3
Difficile iscriversi	1	2	3
I progetti di solito si svolgono lontano da dove vivo	1	2	3
Ci sono poche iniziative disponibili	1	2	3
Non mi sento di possedere le competenze necessarie	1	2	3
Altri mi hanno detto che non ne vale la pena	1	2	3
Sembra complicata	1	2	3
Utilizza una tecnologia obsoleta	1	2	3
Non ho/avevo denaro da spenderci	1	2	3
La mia attrezzatura non è adatta	1	2	3
Il sito del progetto che mi interessa è poco attraente	1	2	3
Non c'erano programmi di formazione disponibili	1	2	3
Il diving non mi ha dato la possibilità	1	2	3
Le informazioni non erano buone e/o erano in un'altra lingua	1	2	3
Altro (specifica):	1	2	3
**2.2. Che cosa ti convincerebbe a partecipare?**			
Sono già convinto/a	1	2	3
Che qualcuno venisse a parlarmene	1	2	3
Aiuto logistico (es. trasporto)	1	2	3
Un incentivo in denaro	1	2	3
Un incentivo come sconti o simile	1	2	3
Un corso di formazione	1	2	3
Materiale informativo nella mia lingua	1	2	3
Una migliore copertura mediatica	1	2	3
Miglior disponibilità di iniziative di Citizen Science	1	2	3
Migliore organizzazione	1	2	3
Tecnologie migliori o nuove	1	2	3
Che il diving mi desse la possibilità di partecipare	1	2	3
Un sito web migliore	1	2	3
Che mi si desse l'attrezzatura necessaria a partecipare	1	2	3
Avere maggiore disponibilità economica	1	2	3
Altro (specifica):	1	2	3
**2.3. Perchè non ti interesserebbe partecipare?**			
Per gli stessi motivi della domanda 1.3	1	2	3
Non ho tempo	1	2	3

Sono troppo giovane/troppo vecchio/a	1	2	3
Ho problemi logistici (es. trasporto)	1	2	3
Sembra difficile	1	2	3
I progetti disponibili si svolgono lontano da dove vivo	1	2	3
Non penso di possedere le competenze necessarie	1	2	3
Non ne traggo benefici	1	2	3
Guadagno troppo poco	1	2	3
Altro (specifica):	1	2	3

2.4. Cosa ti convincerebbe ad interessarti?			
Nulla	1	2	3
Che qualcuno me ne parlasse	1	2	3
Aiuto logistico (es. trasporto)	1	2	3
Un incentivo in denaro	1	2	3
Un incentivo come sconti o altro	1	2	3
Un corso di formazione	1	2	3
Materiale informativo nella mia lingua	1	2	3
Una migliore copertura mediatica	1	2	3
Miglior disponibilità di iniziative di Citizen Science	1	2	3
Migliore organizzazione	1	2	3
Tecnologie migliori o nuove	1	2	3
Un bel sito web	1	2	3
Che il diving mi invitasse a partecipare	1	2	3
Che mi venisse fornita l'attrezzatura necessaria	1	2	3
Avere maggiore disponibilità economica	1	2	3
Altro (specifica):	1	2	3

3. Vuoi aggiungere altro?

Grazie! © Green Bubbles 2015 www.greenbubbles.eu

Il progetto Green Bubbles ha ricevuto un finanziamento dal programma di ricerca e innovazione dell'Unione Europea Horizon 2020, attraverso il contratto Marie Sklodowska-Curie n 643712. Questo documento riflette esclusivamente le opinioni degli autori. La Executive Research Agency non è responsabile per l'uso che può essere fatto delle informazioni in esso contenute.
